# The Past and Future of Fungal Diagnoses in Health Records: A Comparative Analysis and Harmonization of Mycoses ICD Codes

**DOI:** 10.3390/jof12100752

**Published:** 2026-10-08

**Authors:** Marhiah C. Montoya, Brooke D. Esquivel, Mark A. Hoffman, Justin V. Remais, Theodore C. White

**Affiliations:** 1 Division of Biological and Biomedical Systems, School of Science and Engineering, University of Missouri-Kansas City, Kansas City, MO 64110, USA; bdp3k9@umkc.edu; 2 Research Informatics, Children’s Mercy Research Institute, Kansas City, MO 64108, USA; mhoffman@cmh.edu; 3 Division of Environmental Health Sciences, University of California, Berkeley, CA 94720, USA

**Keywords:** International Classification of Diseases, ICD codes, cohort identification, human fungal pathogens, medical mycology, text mining, mycoses, electronic health records

## Abstract

Background: International Classification of Diseases (ICD) codes are used in healthcare for documenting disease and billing, but also for research in epidemiological and translational studies for cohort identification and specimen selection. Periodic code revisions, while necessary, significantly differ in structure and descriptive content, preventing direct cross–version mapping while also complicating use in longitudinal research and coding accuracy during the transition to newer versions. Methods: Mycoses codes within ICD–9, ICD–10, and ICD–11 were manually identified within fungal and non–fungal disease sections using systematic searches for fungal–associated text and eponymous diseases with confirmed fungal etiology. Code descriptions were analyzed to classify etiologic and clinical characteristics for harmonized match scoring. Code similarity was assessed using a heuristically weighted match–score algorithm incorporating classification variables with description–based text mining for clinically relevant semantic harmonization. Codes were categorized into twelve fungal groups (*Aspergillus*, *Blastomyces*, *Candida*, *Coccidioides*, *Cryptococcus*, *Histoplasma*, *Mucorales*, *Paracoccidioides*, *Sporothrix*, Other Fungus–specific Mycoses, Dermatomycoses, and General Mycoses). Bidirectional best–match mapping was performed within each fungal group and manually verified. Results: Fungal codes were organized within and outside the mycoses section in all ICD versions, with several requiring expertise for identification. ICD–9, ICD–10, and ICD–11 contained 94, 117, and 148 mycoses codes accounting for 0.7%, 0.2%, and 0.4% of all codes, respectively. Most codes were taxon-specific (56%, 69%, 61%), but code proportion varied by fungal group and was version-specific. Cross–version bidirectional mapping revealed distinct patterns of code continuity, expansion, and consolidation. Conclusions: A description–based matching approach integrating disease classification and text mining enabled reliable cross–version best–match mapping and produced a resource to improve coding accuracy, support ICD–11 adoption in the United States, and simplify code use in ICD–based studies. Findings emphasize the importance of nomenclature consistency in science and medicine as molecular studies reclassify fungal pathogens.

## 1. Introduction

First published in 1900, the International Classification of Diseases (ICD) is a standardized coding system used in healthcare to describe and record human diseases. This coding system enables communication of health information from patient–level care to billing and supports tracking of regional and national disease trends [1,2]. Since 1948, the ICD has been maintained and revised by the World Health Organization (WHO) as a common language for uniform disease recording, reporting, and monitoring. Over the past five decades, this has resulted in three iterations: ICD–9 (1977), ICD–10 (1992), and ICD–11 (2022) [1,3].

**Figure 1:**
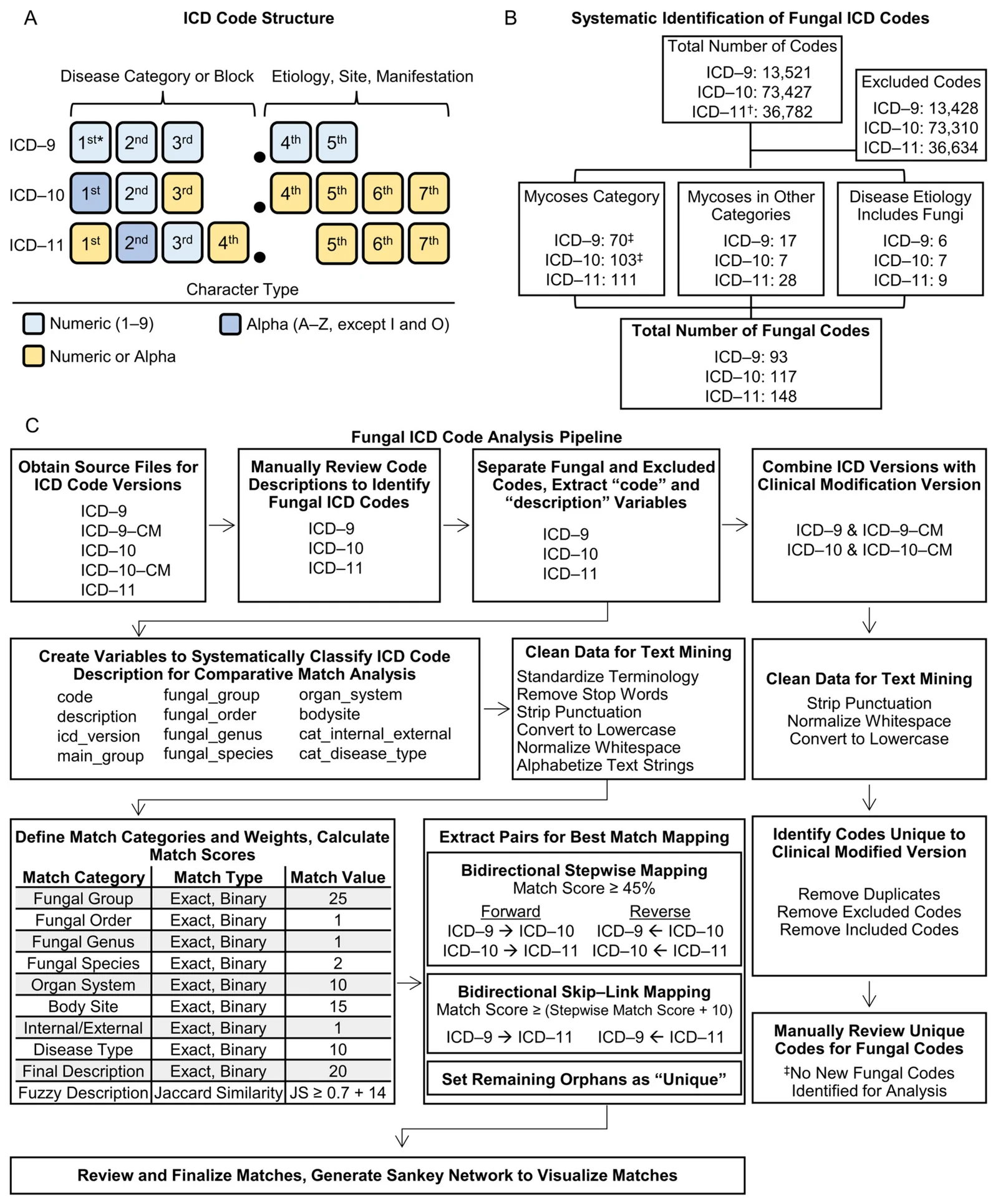
Version–specific code structure, distribution of mycoses codes, and analysis workflow for bidirectional cross-version best–match mapping. (**A**) Code structure is unique to each ICD version. ICD versions differentially incorporate numeric and alpha characters but maintain the hierarchical structure to organize individual codes by disease category before the decimal, and disease etiology, site, or manifestation after. (**B**) Distribution of fungal codes identified across versions, showing initial counts, exclusions, and distribution of fungal codes within and outside the mycoses category. (**C**) ICD code analysis pipeline illustrating the workflow used to classify fungal ICD code descriptions by fungal organism and disease attributes, text processing, match score weights and calculation, and approaches used to identify best cross–version matches were performed bidirectionally across ICD–9, ICD–10, and ICD–11. Clinical modified versions (ICD–9–CM and ICD–10–CM) were evaluated by merging with their base versions, removing duplicate codes, and identifying fungal codes unique to the modified versions. * ICD–9 contains codes with the first place having an E and a “NOINJ” code. ^†^ Includes extension codes and block/chapter identifying code linked to description. ^‡^ Fungal codes identified as non–fungal related due to taxonomic reclassification and codes that did not meet inclusion criteria were excluded.

ICD revisions incorporate major changes to maintain clinical relevance and scientific accuracy. Each version introduces distinct code structures, organizational frameworks, and levels of descriptive granularity (Figure 1A,B). Advancements in medicine, scientific discoveries, and shifts in public health priorities influence decisions to expand, collapse, or redefine codes. These changes can enable more precise documentation of disease etiology, severity, and complexity by reducing the need for multiple codes and improving clinical communication. Revisions can enhance billing accuracy and reimbursement efficiency by facilitating claim review and fraud detection but can also increase the complexity of coding. Adoption of new ICD versions can be delayed when countries implement modified adaptations, as seen with the clinical modification (CM) versions used in the United States (U.S.): ICD–9–CM (1979–2015) and ICD–10–CM (2015–present) [4,5,6]. Though the adoption of updated ICD code versions is essential for healthcare systems, transitions are often challenging due to the significant changes in code structure and descriptions, which often results in under–coding (i.e., use of broad mycoses codes rather than the taxon- or site–specific codes), thereby reducing the intended benefits of newer versions.

Beyond healthcare, ICD codes are widely used in translational and epidemiological research to define study cohorts. In longitudinal studies spanning multiple ICD versions, this process is complicated by ICD revisions and variability in coding practices across providers, institutions, and countries. Studies evaluating ICD coding have demonstrated that these factors can affect the accuracy, validity, and reliability of ICD–based studies [7,8]. In the context of fungal disease, studies consistently report under–coding of mycoses and suggest that reliance on ICD codes alone underestimates disease burden [9,10,11,12,13,14]. Importantly, under–coding is not limited to diseases caused by rare fungi. For example, one candidemia surveillance study found that ~56% (1580/2838) of blood–culture–confirmed cases in the U.S. from 2019 to 2020 did not have a candidiasis–related ICD code documented in their electronic medical records [11]. Furthermore, studies describing the epidemiology of organism–specific mycoses often rely on codes within the mycoses section or those containing taxonomic nomenclature for cohort identification, potentially excluding relevant mycoses codes with eponymous names found outside the mycoses section.

Despite these issues, the increasing availability of large de–identified electronic health record data has created new opportunities for multi–institutional and national epidemiologic studies using datasets like the Oracle Real–World Data platform [15]. In both healthcare and research settings, appropriate use of ICD codes requires a working knowledge of fungal taxonomy, medical mycology, disease etiology, and historical disease terminology. To address these challenges, we developed a cross–version fungal ICD code mapping resource spanning ICD–9, ICD–10, and ICD–11 to support ICD–11 adoption in the U.S. and facilitate accurate cohort identification in fungal research.

## 2. Materials and Methods

### 2.1. Data Sources and Analysis Software

ICD–9 and ICD–10 codes were retrieved from the United States Centers for Medicare and Medicaid Services [16,17], as were ICD–9–CM [18] and ICD–10–CM [19]. We observed that the ICD–10 file from this source included codes unique to the United States ICD–10–CM list. ICD–11 codes were retrieved from the WHO [3,20,21]. ICD code structures underwent comparative analysis to identify similarities and differences (Figure 1A). Further analysis was performed using Microsoft Excel (Version 2608 Build 20326.20142, Redmond, WA, USA) and RStudio (Posit Software R Studio Version 2026.08.2 “Yellow Yarrow”, Boston, MA, USA).

### 2.2. Identification of Mycoses Codes and Semantic Categorization of Disease Descriptions

Codes were reviewed for inclusion if they were in the mycoses category, had fungal etiology, or contained fungal-associated text (“fung-”, “myco-”, “mold”, “mould”), possessives, or fungal nomenclature. Procedure and extension codes were excluded, as were non-fungal codes or those describing toxicity/poisoning due to ingestion. Mycoses codes located within the mycoses category were included in the “Mycoses Category” code counts. Codes identified outside the mycoses category were included in the “Mycoses in Other Categories” count if the description contained taxonomic–specific fungal nomenclature. Codes describing conditions based on occupation, hobbies, or requiring disease–specific etiological knowledge were included in the “Disease Etiology Includes Fungi” category count. Verified mycoses codes were extracted and categorized by organism or clinical themes into twelve fungal groups. Variables capturing fungal taxonomy and disease characteristics were manually created and annotated for each code as part of match score calculation (Figure 1, Table S1). Value assignment for categorical variables classifying taxonomic, etiologic, and clinical characteristics for each ICD code description was performed by the first author, then reviewed by the last author. Any discrepancies were adjudicated by the second author.

### 2.3. Description–Based Text Mining and Match Score Calculation

Code descriptions were standardized and cleaned to reduce content variability for text mining. Comparison pairs were evaluated across the manually curated taxonomic levels (fungal group, order, genus, species), clinical variables (affected organ system, body site, int-/externality, disease type), and final description. Exact matches were binary scored and fuzzy string similarity was calculated for the cleaned description using the Jaccard distance method [22] with a q–gram of two, which allowed for partial matches to range from 0 (no text overlap) to 1 (exact match). A heuristically weighted match–score algorithm was applied, reflecting the relative importance of variables for clinically relevant cross–version mapping and had a maximum value of 100 points (Figure 1C).

### 2.4. Bidirectional Best-Match Mapping and Identification of Version–Specific ICD Codes

Bidirectional best–match mapping was used to identify cross-version relationships among fungal ICD codes and detect version–specific “orphan” codes. Match pairs were filtered to be from the same fungal group but different ICD versions with a minimum cumulative match score of 45 points to prioritize pairs that match on disease manifestation variables while not eliminating pairs that have descriptive inconsistency. Filtered match pairs underwent a standard stepwise mapping strategy where best matches were selected based on the highest cumulative match score, allowing for ties. Match pairs with less than 45 points underwent a skip–link mapping strategy allowing matches between ICD–9 and ICD–11. Skip–link matches were also retained for pairs with match scores equal to or exceeding the stepwise match score by at least 10 points. Codes that were not included in either mapping strategy were “orphan” or version–specific codes. Matches were reviewed (Figure 1C, Table S2), and Sankey network diagrams were constructed using RStudio software to visualize cross–version relationships [23,24].

## 3. Results

### 3.1. Structural Changes in ICD Code Coincide with Expansion of Fungal Disease Codes

Across the three ICD versions analyzed, code structure and organization have undergone significant change. All versions retain a decimal-based hierarchical framework in which characters before the decimal specify the disease category or block and those after specify etiology, site, or manifestation (Figure 1A). ICD–9 exclusively uses numeric characters, whereas ICD–10 introduced an alphanumeric format and expanded code length from five to seven characters. ICD–11 retains the ICD–10 format but has four characters before the decimal rather than three. Both ICD–10 and ICD–11 allow extension codes to provide more granular descriptions; however, they were not included in this study. In conjunction with code instability preventing direct cross–version matching, initial review of each ICD version revealed fungal codes are inconsistently consolidated within the dedicated mycoses section and include eponymous descriptions. Therefore, fungal code identification required a comprehensive manual review of code descriptions, which included a total of 13,521 codes, 73,472 codes, and 36,782 codes for ICD–9, ICD–10, and ICD–11, respectively (Figure 1B). Overall, we identified fungal disease codes within and outside the mycoses section, totaling 94 codes in ICD–9 (70 within, 24 outside), 117 codes in ICD–10 (103 within, 14 outside), and 148 codes in ICD–11 (111 within, 37 outside). Subsequently, each code was categorized as either Taxon–specific Mycoses, if descriptions contained taxonomic nomenclature, or Mycoses with Diverse Fungal Etiology if they did not (Table 1).

### 3.2. Taxonomic Revisions Complicate Taxon–Specific Code Classification While Revealing Differential Expansion Across Fungal Groups

After categorizing codes as *Taxon*–*specific Mycoses* or *Mycoses with Diverse Fungal Etiology*, we quantified code descriptions containing fungus–specific taxonomic nomenclature and found taxon–specific codes comprised approximately 56% (53/94) of ICD–9 fungal codes, 69% (81/117) of ICD–10, and 61% (91/148) of ICD–11 (Table 1). This variation suggests emphasis on organism–specific mycoses was version–specific. To assess organism–specific representation over time, we further categorized each Taxon–specific Mycoses code by fungal organism. This process was complicated by version–specific naming conventions based on fungal sexual state, taxonomic reclassification of organisms as fungal or non–fungal, and variation in taxonomic granularity or medical terminology.

Specifically, the use of teleomorph (sexual) or anamorph (asexual) names was version–specific; however, by ICD–10, most mycoses had adopted use of the anamorph names. For example, the ICD–9 code 117.6—“Allescheriasis [Petriellidosis]” and ICD–10 code B48.2—“Allescheriasis” (teleomorph) were replaced in ICD–11 by 1F2H—“Scedosporiosis” (anamorph) as a result of taxonomic reclassification across genera from *Allescheria boydii* (1922), *Petriellidium boydii* (1970), and *Pseudallescheria boydii* (1982) for the teleomorph form to *Scedosporium apiospermum* for the anamorph form [25].

Taxonomic reclassification also occurred for *Pneumocystis,* which was first considered a protozoan (1909) but was reclassified as a fungus by genetic sequencing in 1988 [26,27]. Conversely, “Rhinosporidosis,” organized within the mycoses section of ICD–9 (117.0) and ICD–10 (B48.1), is caused by *Rhinosporidium seeberi* which was classified as a fungus until 2000 when genetic sequencing reclassified it as protistan parasite [28]. Many fungal organisms have undergone reclassification and renaming between ICD revisions [29]. For example, ICD–10 B48.4—“Penicilliosis” became 1F2K—“Talaromycosis” in ICD–11 reflecting the reclassification of *Penicillium marneffei* to *Talaromyces marneffei* [30]. Importantly, reclassification also affects species–level extension codes for high–priority and reportable fungal pathogens such as *Candidozyma auris* (previously *Candida auris*) [31]. Furthermore, taxonomic granularity was version–specific as observed in *Histoplasma*–related codes specifying species in ICD–9 but reduces specificity in later versions (Table 1, Figures S11 and S12). Likewise, medical terminology varied across versions; for example, “Candidiasis” (ICD–9 and ICD–10) becomes “Candidosis” in ICD–11. Similarly, “Candidiasis of vulva and vagina” (ICD–9 112.1 and ICD–10 B37.3) becomes “Vulvovaginal Candidosis” (1F23.10) in ICD–11. To overcome these challenges, we manually categorized each Taxon–specific Mycoses code by fungal organism and Mycoses with Diverse Fungal Etiology by disease characteristics.

After consolidation, we established 12 fungal groups: (1) *Aspergillus*; (2) *Blastomyces*; (3) *Candida*; (4) *Coccidioides*; (5) *Cryptococcus*; (6) *Histoplasma*; (7) *Mucorales*; (8) *Paracoccidioides*; (9) *Sporothrix;* (10) Other Fungus–specific Mycoses (fungi with <5 codes; *Lacazia*, *Pneumocystis*, *Emergomyces*, *Scedosporium*, *Talaromyces*, *Magnusiomyces*); (11) Dermatomycoses (Dermatophytes, Non–dermatophytes, Nonspecific Dermatomycoses, and *Malassezia*–specific codes to maintain clinical relevance); and (12) General Mycoses (Table 1). This framework revealed code expansion for *Aspergillus*, *Candida*, *Coccidioides*, *Paracoccidioides, Sporothrix*, General Mycoses, Dermatomycoses, and Pneumocystis (within Other Fungus–specific Mycoses), while codes for *Blastomyces*, *Histoplasma*, and *Mucorales* decreased (Table 1). This framework enabled comparative assessment of version–specific representation of fungal groups, while our findings emphasized the need for description–based classification for accurate cross–version code mapping.

### 3.3. Version–Specific Variation in Mycoses Coverage by Fungal Group

**Figure 2:**
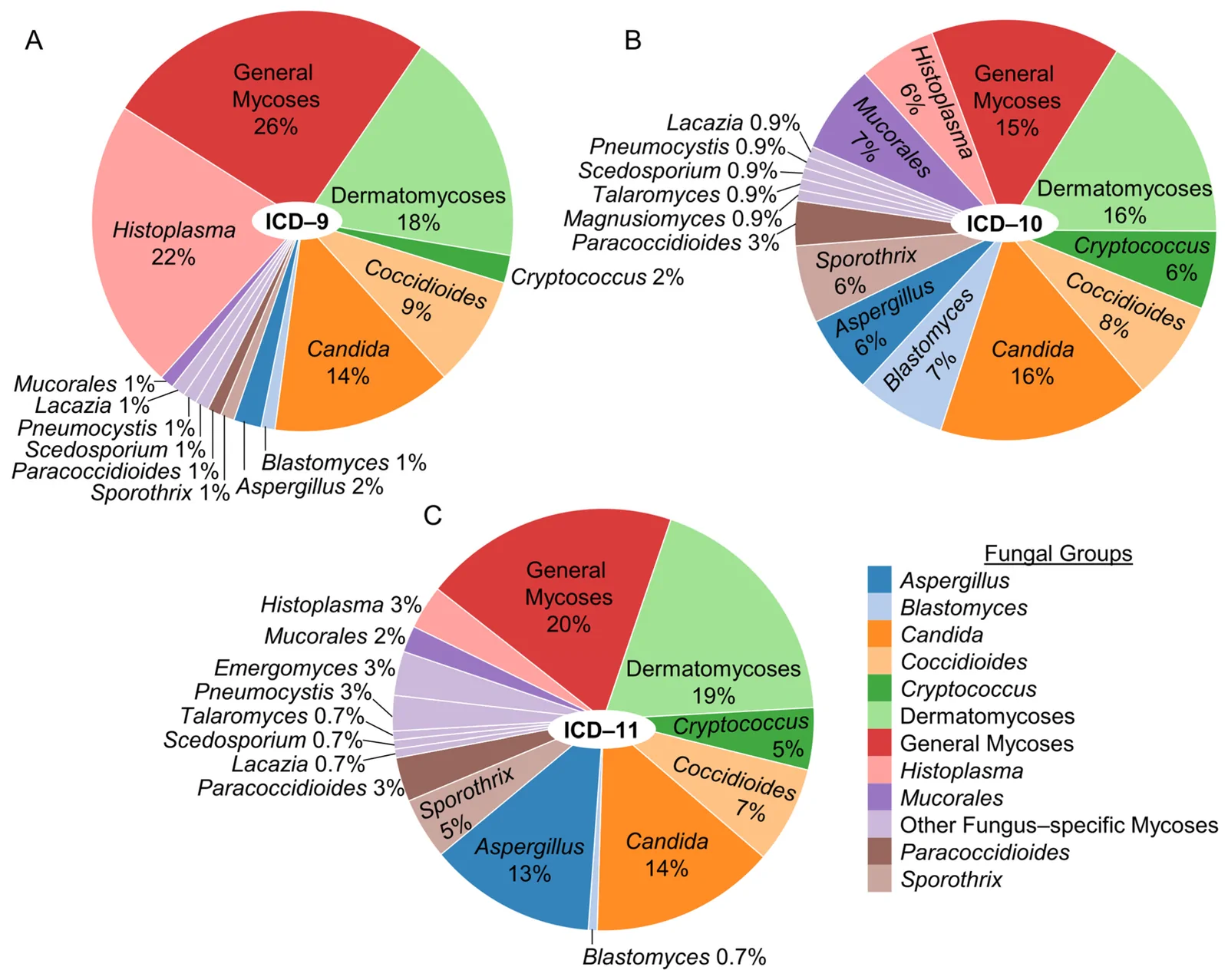
Representation of mycoses by fungal group varies across ICD versions. Proportion of mycoses codes representing each of the twelve fungal groups in (**A**) ICD–9, (**B**) ICD–10, and (**C**) ICD–11. Percentages ≥1% are rounded to the nearest whole number, whereas values <1% are rounded to one decimal place; therefore, displayed percentages may not total 100% exactly.

Across all three ICD versions, there were three fungal groups that consistently contained the greatest proportion of fungal codes: Dermatomycoses, General Mycoses, and *Candida* (Figure 2A–C). From ICD–9 to ICD–11, General Mycoses represented approximately 26% (24/94) in ICD–9, 15% (17/117) in ICD–10, and 20% (29/148) in ICD–11. Likewise, Dermatomycoses represented approximately 18% (17/94), 16% (19/117), and 19% (28/148), and *Candida* represented approximately 14% (13/94), 16% (19/117), and 14% (21/148). Beyond these groups, taxon–specific representation varied across versions.

In ICD–9, taxon-specific coverage was focused on *Histoplasma,* making up ~22% (21/94), which, combined with the top three groups, comprised ~80% (75/94) of fungal codes in ICD–9. The remaining ~20% included approximately 9% *Coccidioides*, 2% *Aspergillus*, 2% *Cryptococcus*, 3% Other Fungus–specific Mycoses (~1% *Lacazia*, ~1% *Pneumocystis*, ~1% *Scedosporium*), and ~1% each of *Blastomyces*, *Mucorales*, *Paracoccidioides*, and *Sporothrix* (Table 1, Figure 2A). This distribution suggests greater emphasis on broad mycoses compared to organism–specific mycoses, likely reflecting morphology–based diagnostic constraints from the 1970s–1990s [32]. For example, molecular diagnostics such as the polymerase chain reaction technique was developed in the 1980s [33], but its adaptation and use for clinical diagnostics occurred between the 1990s–2000s [34]. Likewise, clinical antigen detection assays began in the 1960s for *Cryptococcus* species [35] and expanded between the 1970s–1980s to include fungi such as *Aspergillus* [36]. However, though diagnostic limitations may affect the detection and differentiation of fungal species, whether they directly contributed to differences in the representation of mycoses across ICD versions remains uncertain.

In ICD–10, General Mycoses, Dermatomycoses, and *Candida* together represented 47% (55/117) of all fungal codes while the remaining 53% had a more balanced distribution with approximately 8% *Coccidioides*, 7% *Blastomyces*, 7% *Mucorales*, 6% *Aspergillus*, 6% *Cryptococcus*, 6% *Histoplasma*, 6% *Sporothrix*, 3% *Paracoccidioides*, and 4% Other Fungus–specific Mycoses (~0.9% *Lacazia*, ~0.9% *Pneumocystis*, ~0.9% *Scedosporium*, ~0.9% *Magnusiomyces*, ~0.9% *Talaromyces*) reflecting broader taxon–specific representation (Table 1, Figure 2B). ICD–10 added codes for mycoses caused by *Talaromyces* and *Magnusiomyces*.

In ICD–11, the taxon–specific focus shifted to *Aspergillus* making up ~13% (19/148) and, combined with General Mycoses, Dermatomycoses, and *Candida*, account for ~66% (97/148) of fungal codes (Table 1, Figure 2C). The remaining ~34% include approximately 7% *Coccidioides*, 5% *Cryptococcus*, 5% *Sporothrix*, 3% *Histoplasma*, 3% *Paracoccidioides*, 2% *Mucorales*, 0.7% *Blastomyces*, and 8% Other Fungus–specific Mycoses (3% *Pneumocystis*, 3% *Emergomyces*, and ~0.7% *Lacazia*, ~0.7% *Scedosporium*, ~ 0.7% *Talaromyces*). ICD–11 also eliminated the *Magnusiomyces* code and added five *Emergomyces* codes. This shift in code distribution corresponds with increased focus on organisms listed as WHO priority fungal pathogens [37].

**Figure 3:**
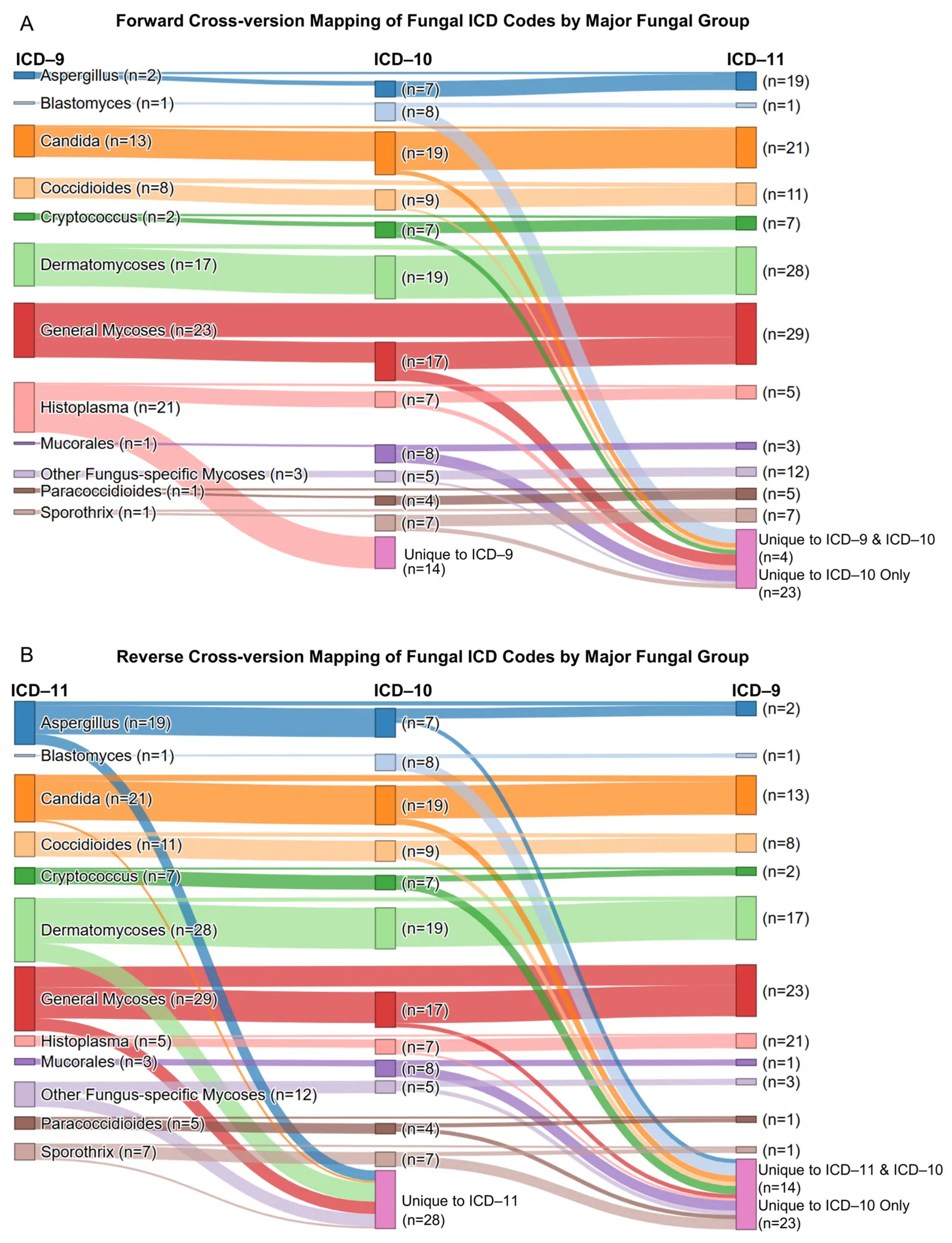
Bidirectional mapping of fungal ICD codes reveals taxon-specific expansion and collapse of disease coverage. Sankey network diagrams of the (**A**) forward and (**B**) reverse cross–version best-match mapping of ICD codes by fungal group visualizing the expansion and collapse of mycoses representation among the twelve fungal groups. Columns represent ICD version, nodes represent fungal groups, and links represent one or more code match pair(s).

Match analysis of standardized descriptions between codes from the same fungal group isolated the “best–match” (highest) score for each match pair (Table S2) and mapped matches in forward (Figure 3A) and reverse (Figure 3B) Sankey network diagrams. Forward mapping revealed 14 *Histoplasma* codes unique to ICD–9 (Figure 3A and Figure S11). In contrast, the 27 codes that were either unique to ICD–10 or had continuity between ICD–9 and ICD–10 stemmed from all groups except *Aspergillus*, *Paracoccidioides*, and Dermatomycoses. Reverse mapping revealed 28 codes unique to ICD–11 from *Aspergillus*, *Candida*, *Sporothrix*, Other Fungus–specific Mycoses, Dermatomycoses, and General Mycoses. Reverse mapping also revealed 14 codes with continuity between ICD–10 and ICD–11 from all groups except *Blastomyces*, *Histoplasma*, *Mucorales*, and Dermatomycoses, and 23 codes unique to ICD–10 from all fungal groups except *Aspergillus*, *Paracoccidioides*, and Dermatomycoses (Figures S1–S24). These patterns highlight version–specific expansion and consolidation, requiring further investigation to determine how code descriptions changed across ICD versions.

### 3.4. Directional Mapping Reveals Partial Continuity and Prioritization of Specific Fungal Diseases

To determine how individual fungal groups contributed to the expansion and collapse of fungal ICD codes across versions, bidirectional best–match mapping was performed for each fungal group (Figures S1–S24). Two distinct patterns emerged: (1) fungal groups that were preserved with moderate changes or consistent expansion and (2) groups that underwent expansion followed by consolidation or collapse.

**Figure 4:**
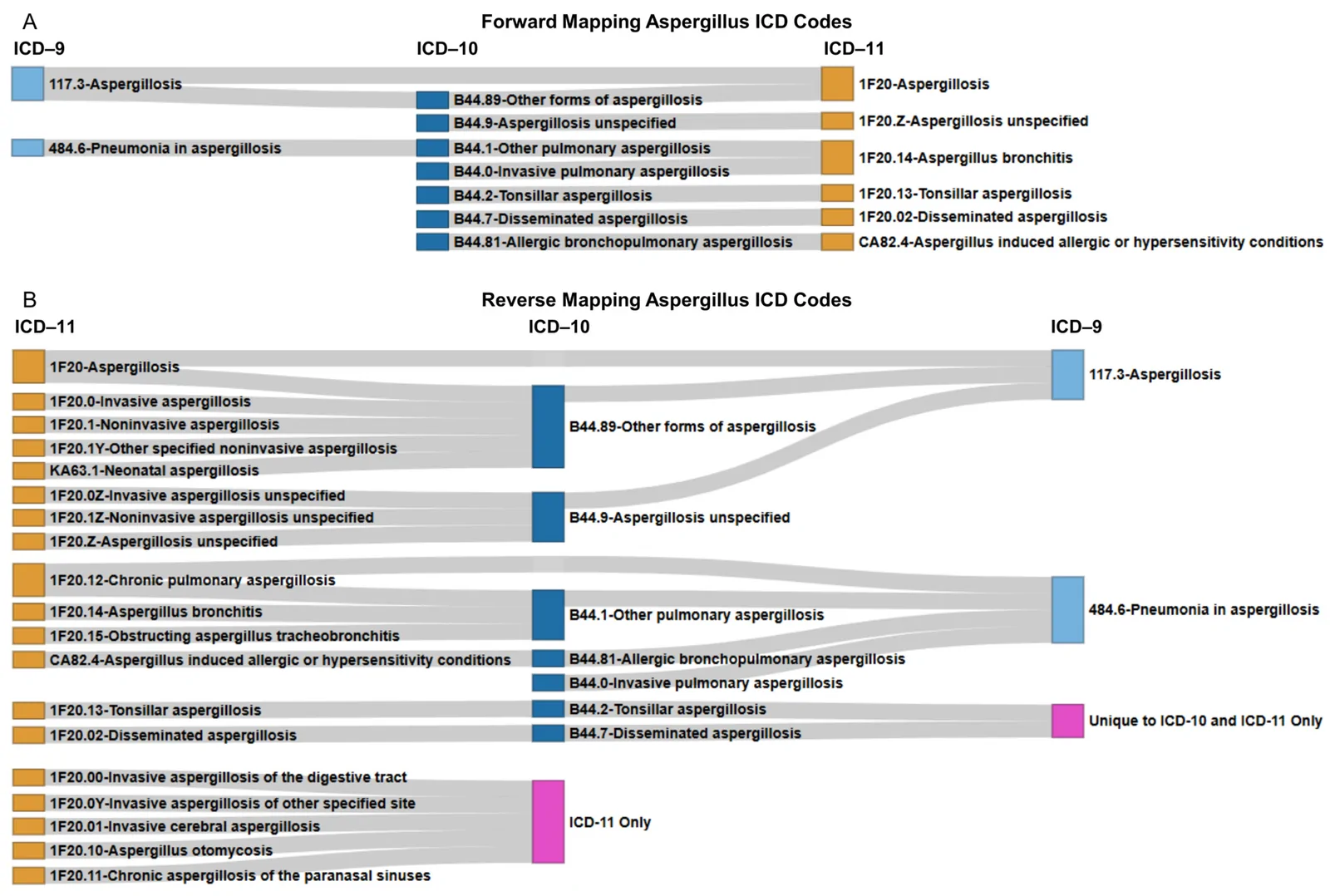
Forward and reverse mapping of *Aspergillus* ICD codes. Forward (**A**) and reverse (**B**) Sankey network diagrams show best–match mapping of *Aspergillus* ICD codes. Each node column represents a different ICD version, each node represents a different ICD code, and each link represents one fungal code match pair.

Exemplifying the first pattern, the bidirectional mapping of *Aspergillus* codes illustrates cross–version continuity and preservation of codes in the forward code mapping (Figure 4A), while reverse mapping illustrates the expansion of codes in ICD–11 and how descriptive granularity is reduced in ICD–10 and ICD–9 (Figure 4B). Overall, the expansion of aspergillosis codes can primarily be attributed to increased emphasis on pulmonary airway disease, invasiveness, frequency of disease, and the addition of new sites of infection (Table 2). Taken together, bidirectional mapping of *Aspergillus* codes reflects a pattern of incremental expansion and preservation of codes from earlier ICD versions. Other fungal groups exhibiting code preservation with moderate changes or consistent expansion included *Candida*, *Coccidioides*, *Paracoccidioides*, Other Fungus–specific Mycoses, Dermatomycoses, and General Mycoses. *Candida* codes were consistently preserved, and expansion of site–specific candidiasis codes included increased emphasis on skin conditions. *Coccidioides* codes were preserved across ICD versions but expanded to include codes describing extra–thoracic disease. *Paracoccidioides* codes expanded through the addition of codes for respiratory, systemic, and nonspecific disease. Other Fungus–specific Mycoses codes from ICD–9 were preserved across all versions, and expansion within this group was a result of the addition of new taxon–specific codes for conditions caused by *Talaromyces* and *Emergomyces* with increased emphasis on pulmonary diseases. Dermatomycoses expansion was driven by an increase in codes for nonspecific dermatomycoses and dermatomycoses caused by non–dermatophytes. General mycoses initially showed a reduction in codes related to musculoskeletal conditions but increased codes for nervous and respiratory diseases while also adding new codes of mycoses affecting the digestive tract, systemic infections, and pediatric infections. Overall, these groups demonstrate consistent code preservation and expansion of codes specifying details of disease manifestation.

Conversely, the second pattern can be observed for *Histoplasma*, where the forward code mapping displays a significant reduction in species–specific codes from earlier ICD versions compared to ICD–11, emphasizing the loss of descriptive granularity (Figure S11), while the reverse highlights the preserved codes (Figure S12). Other fungal groups that underwent expansion followed by consolidation or collapse included *Blastomyces*, *Cryptococcus*, *Mucorales*, and *Sporothrix*. *Blastomyces* expanded in ICD–10 by increasing the coverage of affected body sites but were consolidated back down to one code in ICD–11. *Cryptococcus* codes focused on systemic, nervous, and respiratory diseases, and expansion included the addition of cutaneous (B45.2) and osseous (B45.3) cryptococcosis codes that were eliminated in ICD–11. *Mucorales* underwent expansion in ICD–10 with the addition of codes specifying new body sites and fungal etiology, but ultimately only the latter were preserved. *Sporothrix* expansion added new body sites and preserved codes focused on pulmonary, invasive, and nonspecific diseases. Collectively, these fungal groups demonstrate the overall pattern of a short-lived expansion of site-specific codes that was not preserved in ICD–11.

## 4. Study Limitations

Study limitations, or rather, methodological considerations that can affect the interpretation of these findings primarily relate to the manual aspects of the methods used in this study. First, the manual identification of mycoses ICD codes, categorization of disease etiology and characteristics, and assignment of weights of each variable involved in the calculation of match–scores introduces the potential for subjective classification and limits reproducibility. Manual identification of mycoses codes was necessary due to inconsistent consolidation of mycoses within the dedicated mycoses section, the existence of diseases with multiple etiologies, and variability in disease descriptions. In an attempt to reduce the effects of limitations, our methods required that decisions relating to the categorization variables, value assignments, and weights used in our match-score algorithm were evaluated by multiple subject matter experts with decades of experience in medical mycology, fungal epidemiology, and healthcare practices. In addition, variable weights and the minimum match–score threshold were developed heuristically with careful consideration to ensure that final matches were both taxonomically accurate, clinically relevant, and semantically precise, rather than representing mathematically optimized or independently validated thresholds. Importantly, this study provides the code used to perform this analysis so that weights and thresholds can be modified for individual use. Overall, the match–score algorithm and cross–version matches developed in this study were designed to prioritize clinical relevance for mycoses ICD codes and, in some instances, provide greater specificity than the WHO cross–version mapping tables; however, these differences do not constitute greater accuracy without additional independent validation studies [38]. For example, in both WHO ICD–10/ICD–11 Mapping Tables that map ICD–10 codes to either one or multiple ICD–11 codes, the ICD–10 code B35.0—“Tinea barbae and tinea capitis” which describes a dermatomycosis condition of the beard and scalp, only maps to the ICD–11 code 1F28.Z—“Dermatophytosis, unspecified” rather than suggesting multiple potential matches including the clinically relevant code 1F28.0—“Dermatophytosis of the scalp” along with the currently suggested match. Several other examples like this were found, highlighting the necessity of this study as well as the need for a visual representation of cross–version matches for ease of use in daily clinical practice and medical coding.

Second, this study excluded ICD extension codes from analysis, which may limit the apparent diagnostic granularity and should be considered when interpreting the apparent loss or consolidation of diagnostic specificity across code versions. Extension codes can provide additional specificity regarding clinical manifestations and the etiologic fungal species; however, they do not increase the number of ICD codes dedicated for the documentation of specific mycoses or mycoses caused by specific fungal pathogens. Finally, some codes included in this study represent conditions that can have fungal or non-fungal etiologies (e.g., ICD–9 39.4—“Madura foot”) and allergic hypersensitivity diseases such as Farmer’s Lung (ICD–9 495.0, ICD–10 J67.0, ICD–11 CA70.0), which is an allergic hypersensitivity pneumonitis to moldy organic material (hay, grain, straw, compost, etc.) and can result in lung damage for severe or chronic cases. Though such codes do not represent diseases that are exclusively caused by fungal pathogens or explicitly indicate the disease was caused by fungal growth in human tissues, they were retained because of their clinical importance and underrepresentation in epidemiological studies of fungal diseases and diseases induced by exposure to fungal pathogens. The inclusion of such disease codes may inflate the representation of fungal diseases included in each ICD version; however, to mitigate this potential inflation, we separated these disease codes from taxon–specific mycoses codes to distinguish them from diseases caused exclusively by fungal pathogens.

## 5. Discussion

Successive ICD versions reflect evolving public health priorities and scientific advancements, but revisions are accompanied by variation in code structure and description text, preventing direct cross-version mapping. This study presents the first comprehensive analysis of mycoses codes across ICD–9, ICD–10, and ICD–11, providing a cross–version reference map of mycoses codes supporting the future U.S. transition to ICD–11 for clinicians and medical coders but also improving case identification for researchers.

Although each revision expanded mycoses codes, important gaps persist that affect accurate representation of a patient’s clinical history as well as case identification. For instance, “Chronic Mucocutaneous Candidiasis” (ICD–11 1F23.14) is a genetic disorder affecting the immune system that results in chronic *Candida* infections and, despite being studied since the 1970s, did not have a code until ICD–11 [39]. Such instances highlight how new codes can improve specificity for clinical communication and how cohort identification using new codes can be difficult for longitudinal studies. ICD–based cohort identification can also miss cases when studies only use codes within the mycoses section despite multiple diseases with eponymous names having a fungal etiology. This includes diseases like Malt worker’s lung (ICD–9 49.54, ICD–10 J67.4, ICD–11 CA70.4) which is caused by *Aspergillus clavatus,* Bird fancier’s lung (ICD–10 J67.2, ICD–11 CA70.2) which can be caused by multiple fungal organisms, or diseases caused by both bacteria and fungi (e.g., ICD–9 39.4—“Madura foot”, ICD–10 B47.9—“Mycetoma unspecified”). Furthermore, coding accuracy is also influenced by training and experience when medical terminology clashes with fungal taxonomy. For example, prior to being renamed, Paracoccidioidomycosis was previously referred to as “South American Blastomycosis,” causing understandable confusion, as it was coded in ICD–9 as 116.0—“Blastomycosis” rather than *Paracoccidioides* is accurately coded in as 116.1—“Paracoccidioidomycosis”.

Our findings emphasize the importance of using accurate and current fungal nomenclature consistently in scientific and medical literature to reduce confusion as accessibility and advancements in molecular techniques enable more accurate taxonomic reclassification of fungal pathogens. Findings also identified gaps in species–level representation for many fungal organisms, including those that have been categorized by the WHO as priority fungal pathogens [37]. Infections caused by different fungal species can vary in clinical presentation, severity, transmission, and therapeutic response, but this information is lost without species–specific ICD codes. In contrast to many bacterial species that have different ICD codes in ICD–10, such as *Streptococcus* Group A (40.0) and Group B (40.1) or *Brucella* species *B. melitensis* (A23.0), *B. abortus* (A23.1), *B. suis* (A23.2), or *B. canis* (A23.3), fungal species do not have species–specific ICD codes, including many that are frequently resistant to antifungals. Many current and former *Candida* species have been associated with antifungal resistance, including *Candida albicans*, *Candida glabrata* [40], *Pichia kudriavzevii* (previously *Candida krusei*) [41], *Candida parapsilosis*, and *Candidozyma auris* (previously *Candida auris*) [31,42,43]. Similarly, *Cryptococcus gattii* is a distinct species from *Cryptococcus neoformans* that can infect immune–competent individuals but lacks species-specific codes [44]. The importance and advantages of using species–level information that is up–to–date with scientific advancements apply to many fields but require those within the scientific and medical communities to stay current with novel findings and appropriately integrate taxonomic nomenclature changes in future work. For public health initiatives, species–level identification of the infecting fungus could support outbreak investigations and response by facilitating early detection and reporting of notifiable pathogens to public health agencies. For research, species–level identification of fungal pathogens could improve inference of epidemiological trends of mycoses caused by emerging or high–risk organisms while also enabling better targeting of funding towards high–priority fungal species or sub–species.

Together, these findings highlight that reliance on ICD codes alone in clinical practice limits the accurate representation of disease manifestation and, in research, can result in the underestimation of fungal disease burden [9,10,11,12]. Improving coding accuracy will require the adoption of ICD–11 in the U.S. and use of extension codes to further increase specificity. This cross–version mapping of mycoses codes represents a step towards improving coding accuracy, but further advances will likely reform coding practices as many groups are already training and testing the use of AI natural language processing and machine learning to enhance coding accuracy through automation or improved coding processes that assist clinicians and medical coders [12,45,46,47,48,49,50].

## Figures and Tables

**Table 1:** Quantification of mycoses codes by fungal group and ICD version. The number of fungal codes within each ICD version was quantified and categorized into twelve fungal groups (bold text) and subgroups (normal text). Change in the number of ICD codes is visualized in the trend column.Trend key defines each trend type where consecutive increase is represented by a yellow cell containing two plus signs (+ +); single increase is an orange cell with a single plus sign (+); decrease, increase is a pink cell with a minus sign and a plus sign (− +); increase, decrease is a purple cell with a plus sign and a minus sign (+ −); single decrease is a blue cell with a minus sign (−); consecutive decrease is a grey cell with two minus signs (− −); and no change is a white blank cell.

		ICD Code Version						
	Fungal Group Subgroup	ICD-9	ICD-10	ICD-11	Trend			Trend Key
**Taxon–specific Mycoses**	***Aspergillus***	**2**	**7**	**19**	+ +		+ +	Consecutive Increase
	***Blastomyces***	**1**	**8**	**1**	+ −		+	Single Increase
	***Candida***	**13**	**19**	**21**	+ +		− +	Decrease, Increase
	***Coccidioides***	**8**	**9**	**11**	+ +		+ −	Increase, Decrease
	***Cryptococcus***	**2**	**7**	**7**	+		−	Single Decrease
	***Histoplasma***	**21**	**7**	**5**	− −		− −	Consecutive Decrease
	*Histoplasma*	7	1	3	− +			No Change
	*Histoplasma capsulatum*	7	5	1	− −			
	*Histoplasma duboisii*	7	1	1	−			
	***Mucorales***	**1**	**8**	**3**	+ −			
	***Paracoccidioides***	**1**	**4**	**5**	+ +			
	***Sporothrix***	**1**	**7**	**7**	+			
	**Other Fungus–specific Mycoses**	**3**	**5**	**12**	+ +			
	*Emergomyces*	0	0	5	+			
	*Magnusiomyces*	0	1	0	+ −			
	*Lacazia*	1	1	1				
	*Talaromyces*	0	1	1	+			
	*Pneumocystis*	1	1	4	+			
	*Scedosporium*	1	1	1				
**Mycoses with Diverse Fungal Etiology**	**Dermatomycoses**	**17**	**19**	**28**	+ +			
	Dermatophyte	10	10	11	+			
	*Malassezia*	1	1	3	+			
	Nondermatophyte	4	6	7	+ +			
	Nonspecific Dermatomycoses	2	2	7	+			
	**General Mycoses**	**24**	**17**	**29**	− +			
	Nonspecific	2	3	7	+ +			
	Occupation–related	6	7	7	+			
	Pigmented Fungi	2	3	2	+ −			
	Nonspecific Arthromycoses	10	0	2	− +			
	Nonspecific Neuromycoses	1	0	4	− +			
	Nonspecific Oculomycoses	1	4	0	+ −			
	Nonspecific Otomycoses	1	0	1	− +			
	Nonspecific Pediatric Mycoses	0	0	3	+			
	Nonspecific Pneumomycoses	1	0	3	− +			
	**Grand Total**	**94**	**117**	**148**	+ +			

**Table 2:** Distribution of mycoses codes by fungal group and organ system across ICD versions. Fungal disease codes for the twelve fungal groups are categorized by organ systems across ICD–9, ICD–10, and ICD–11. Code expansion is visualized by a color scale to emphasize the variation in the number of codes across ICD versions and body site of disease.

		Fungal Group																																			
		*Aspergillus*			*Blastomyces*			*Candida*			*Coccidioides*			*Cryptococcus*			*Histoplasma*			*Mucorales*			*Paracoccidioides*			*Sporothrix*			Other Fungus–specific Mycoses			Dermatomycoses			General Mycoses		
	ICD Version	9	10	11	9	10	11	9	10	11	9	10	11	9	10	11	9	10	11	9	10	11	9	10	11	9	10	11	9	10	11	9	10	11	9	10	11
Organ System	Cardiovascular							1	1								6																				
	Digestive			1				3	3	2											1															1	3
	Extra–thoracic										1		3																								
	Genitourinary							2	6	2		1																									
	Immune		1	1																																	
	Integumentary					1		1	2	7		1	1		1						1							1				17	19	25			
	Lymphatic		1	1																							1	1									
	Musculoskeletal														1												1								10		2
	Nervous			2		1		2	2	2	1	1	1	1	1	2	6				1						1								3	5	5
	Respiratory	1	2	4		3		1	1	1	3	3	3		1	1	3	3	1		1			1	1		1	1			3				7	7	10
	Systemic		1	1		1		1	1	3	1	1	1		1	1		1			1			1	1		1	1			1			1			1
	Pediatric			1					1	1																								2			3
	Nonspecific	1	2	8	1	2	1	2	2	3	2	2	2	1	2	3	6	3	4	1	3	3	1	2	3	1	2	3	3	5	8				4	4	5
	Total Codes	2	7	19	1	8	1	13	19	21	8	9	11	2	7	7	21	7	5	1	8	3	1	4	5	1	7	7	3	5	12	17	19	28	24	17	29
																																		
	Number of Codes				1	2	3	4	5	6	7	≥8																									

## Data Availability

All coding, data files, figures, and tables can be found at https://github.com/marhiahcmontoya/Fungal-ICD-Code-Mapping-Project (access date: 30 September 2026).

## Supplementary Materials

The following supporting information can be downloaded at https://www.mdpi.com/article/10.3390/jof12100752/s1: Figures S1–S24: Bidirectional Best-Match ICD Code Mapping of Each Fungal Group; Table S1: Systematic Classification of Mycoses ICD Codes; Table S2: Best-Match Scores for Mapped ICD Code Match-Pairs; Supplemental Methods: Supplemental Methods of Analysis Pipeline.

## Abbreviations

The following abbreviations are used in this manuscript:

ICD	International Classification of Diseases
CM	Clinical Modification
WHO	World Health Organization
U.S.	United States
