# Supplementary material for: The Past and Future of Fungal Diagnoses in Health Records: A Comparative Analysis and Harmonization of Mycoses ICD Codes
Source: J Fungi (Basel). 2026 Oct 8;12(10):752. doi: 10.3390/jof12100752 (PMC13706049; doi:10.3390/jof12100752)
Supplement: Supplementary file 1 [file jof-12-00752-s001.zip › Figure_S1-S24_BestMatchMapping_Final.pdf]

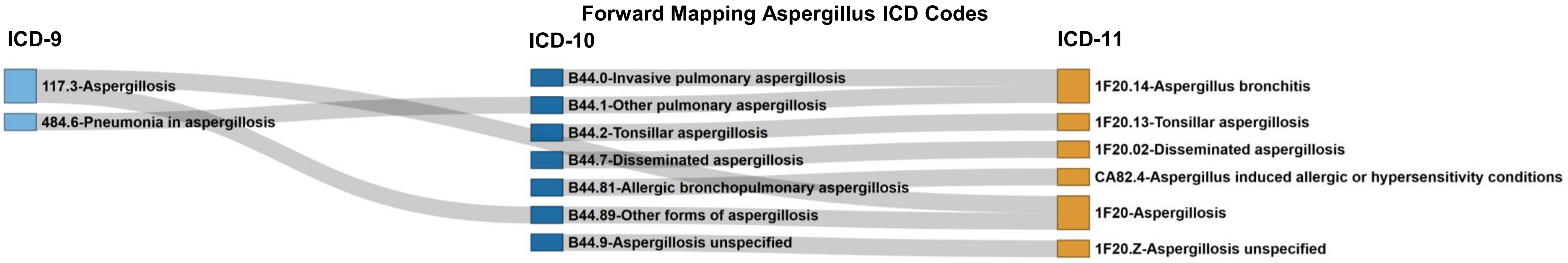

Supplemental Figure S1. Aspergillus ICD Code Mapping: Best Match Forward Sankey Network

Nodes represent individual ICD codes across versions (ICD-9, -10, -11), connected by links indicating mapping pairs with the highest match score, with ties. Primary mapping utilized a stepwise strategy requiring a minimum cumulative match score of 45 out of 100 points. To capture complex relationships, a "skip-link" strategy was employed for pairs that either failed the initial threshold or exceed the stepwise score by ≥ 10 points. Codes with unlinked nodes ("Orphan" codes) represent version-specific entries that did not meet the minimum match criteria in either strategy and are indicated by a pink node with text indicating the ICD version(s) to which it is unique. Light Blue Nodes: ICD-9, Dark Blue Nodes: ICD-10, and Yellow Nodes: ICD-11

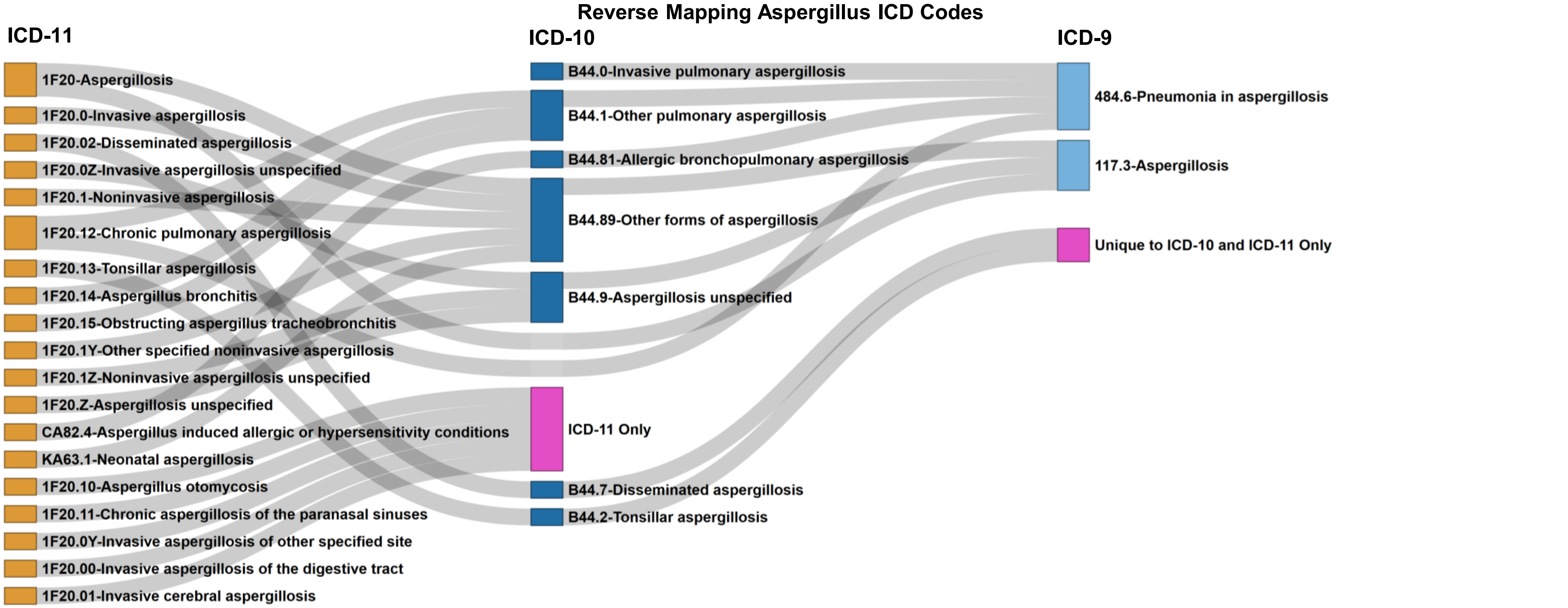

Supplemental Figure S2. Aspergillus ICD Code Mapping: Best Match Reverse Sankey Network

Nodes represent individual ICD codes across versions (ICD-11, -10, -9), connected by links indicating mapping pairs with the highest match score, with ties. Primary mapping utilized a stepwise strategy requiring a minimum cumulative match score of 45 out of 100 points. To capture complex relationships, a "skip-link" strategy was employed for pairs that either failed the initial threshold or exceed the stepwise score by ≥ 10 points. Codes with unlinked nodes ("Orphan" codes) represent version-specific entries that did not meet the minimum match criteria in either strategy and are indicated by a pink node with text indicating the ICD version(s) to which it is unique. Light Blue Nodes: ICD-9, Dark Blue Nodes: ICD-10, and Yellow Nodes: ICD-11

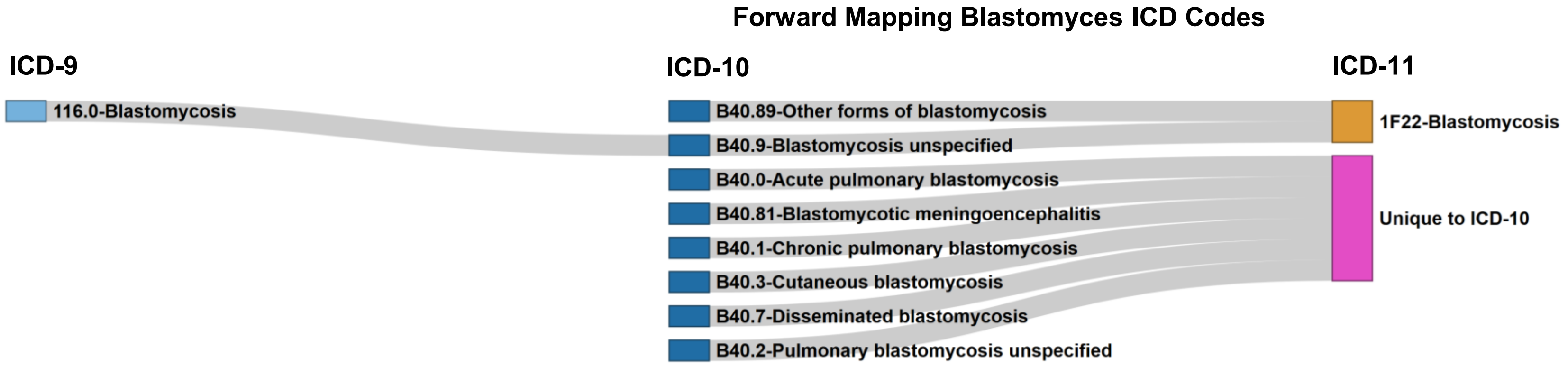

Supplemental Figure S3. Blastomyces ICD Code Mapping: Best Match Forward Sankey Network
Nodes represent individual ICD codes across versions (ICD-9, -10, -11), connected by links indicating mapping pairs with the highest match score, with ties. Primary mapping utilized a stepwise strategy requiring a minimum cumulative match score of 45 out of 100 points. To capture complex relationships, a "skip-link" strategy was employed for pairs that either failed the initial threshold or exceed the stepwise score by ≥ 10 points. Codes with unlinked nodes ("Orphan" codes) represent version-specific entries that did not meet the minimum match criteria in either strategy and are indicated by a pink node with text indicating the ICD version(s) to which it is unique. Light Blue Nodes: ICD-9, Dark Blue Nodes: ICD-10, and Yellow Nodes: ICD-11

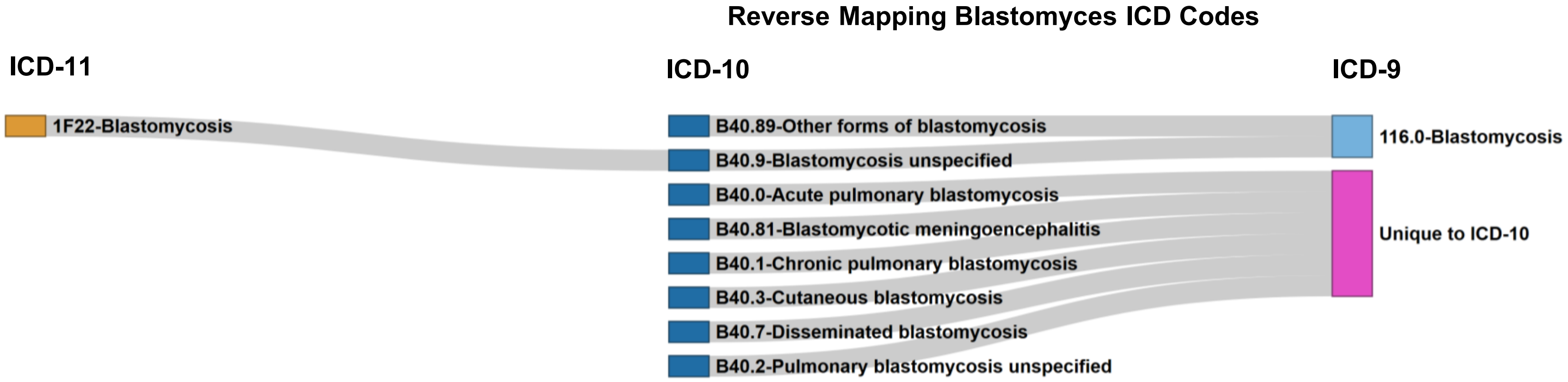

Supplemental Figure S4. Blastomyces ICD Code Mapping: Best Match Reverse Sankey Network
Nodes represent individual ICD codes across versions (ICD-11, -10, -9), connected by links indicating mapping pairs with the highest match score, with ties. Primary mapping utilized a stepwise strategy requiring a minimum cumulative match score of 45 out of 100 points. To capture complex relationships, a "skip-link" strategy was employed for pairs that either failed the initial threshold or exceed the stepwise score by ≥ 10 points. Codes with unlinked nodes ("Orphan" codes) represent version-specific entries that did not meet the minimum match criteria in either strategy and are indicated by a pink node with text indicating the ICD version(s) to which it is unique. Light Blue Nodes: ICD-9, Dark Blue Nodes: ICD-10, and Yellow Nodes: ICD-11

Forward Mapping Candida ICD Codes

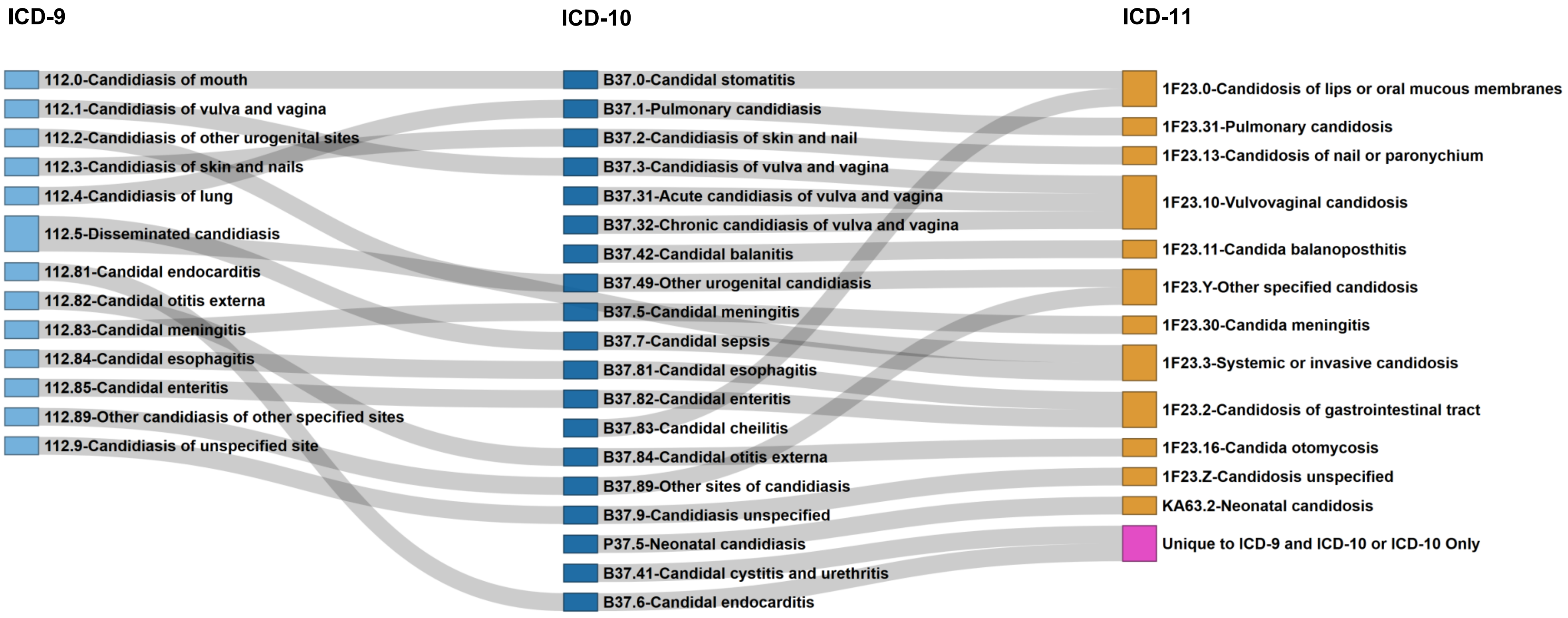

Supplemental Figure S5. Candida ICD Code Mapping: Best Match Forward Sankey Network
Nodes represent individual ICD codes across versions (ICD-9, -10, -11), connected by links indicating mapping pairs with the highest match score, with ties. Primary mapping utilized a stepwise strategy requiring a minimum cumulative match score of 45 out of 100 points. To capture complex relationships, a “skip-link” strategy was employed for pairs that either failed the initial threshold or exceed the stepwise score by ≥ 10 points. Codes with unlinked nodes (“Orphan” codes) represent version-specific entries that did not meet the minimum match criteria in either strategy and are indicated by a pink node with text indicating the ICD version(s) to which it is unique. Light Blue Nodes: ICD-9, Dark Blue Nodes: ICD-10, and Yellow Nodes: ICD-11

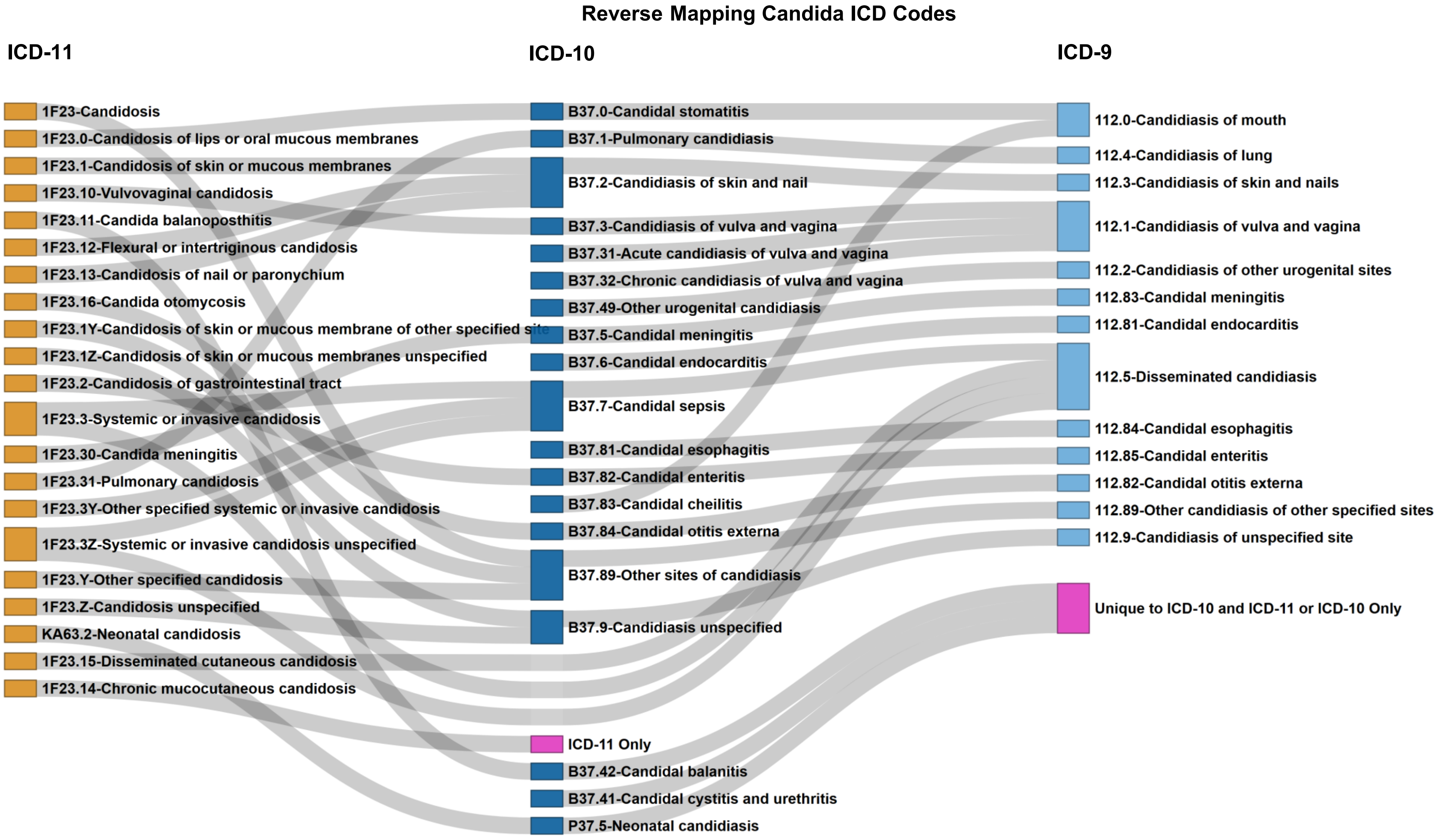

Supplemental Figure S6. Candida ICD Code Mapping: Best Match Reverse Sankey Network
Nodes represent individual ICD codes across versions (ICD-11, -10, -9), connected by links indicating mapping pairs with the highest match score, with ties. Primary mapping utilized a stepwise strategy requiring a minimum cumulative match score of 45 out of 100 points. To capture complex relationships, a "skip-link" strategy was employed for pairs that either failed the initial threshold or exceed the stepwise score by ≥ 10 points. Codes with unlinked nodes ("Orphan" codes) represent version-specific entries that did not meet the minimum match criteria in either strategy and are indicated by a pink node with text indicating the ICD version(s) to which it is unique. Light Blue Nodes: ICD-9, Dark Blue Nodes: ICD-10, and Yellow Nodes: ICD-11

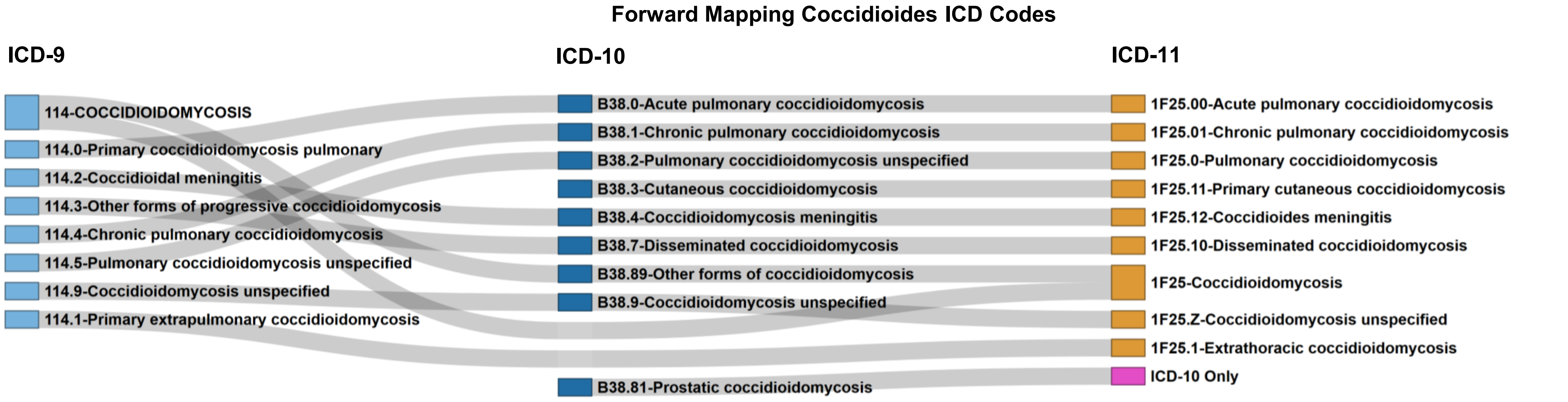

Supplemental Figure S7. Coccidioides ICD Code Mapping: Best Match Forward Sankey Network
Nodes represent individual ICD codes across versions (ICD-9, -10, -11), connected by links indicating mapping pairs with the highest match score, with ties. Primary mapping utilized a stepwise strategy requiring a minimum cumulative match score of 45 out of 100 points. To capture complex relationships, a "skip-link" strategy was employed for pairs that either failed the initial threshold or exceed the stepwise score by ≥ 10 points. Codes with unlinked nodes ("Orphan" codes) represent version-specific entries that did not meet the minimum match criteria in either strategy and are indicated by a pink node with text indicating the ICD version(s) to which it is unique. Light Blue Nodes: ICD-9, Dark Blue Nodes: ICD-10, and Yellow Nodes: ICD-11

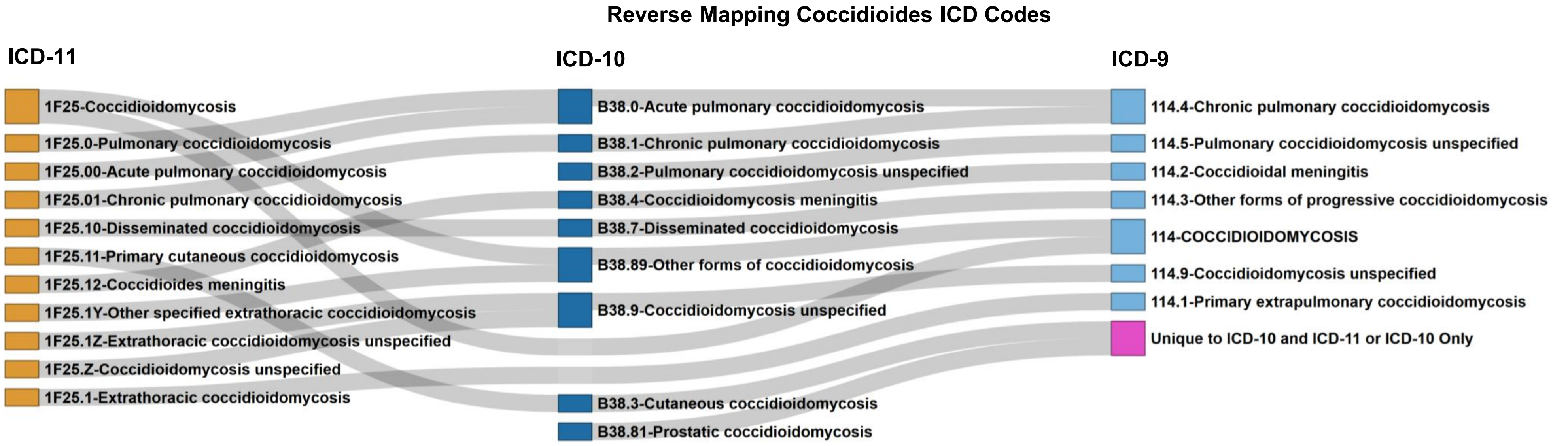

Supplemental Figure S8. Coccidioides ICD Code Mapping: Best Match Reverse Sankey Network
Nodes represent individual ICD codes across versions (ICD-11, -10, -9), connected by links indicating mapping pairs with the highest match score, with ties. Primary mapping utilized a stepwise strategy requiring a minimum cumulative match score of 45 out of 100 points. To capture complex relationships, a "skip-link" strategy was employed for pairs that either failed the initial threshold or exceed the stepwise score by ≥ 10 points. Codes with unlinked nodes ("Orphan" codes) represent version-specific entries that did not meet the minimum match criteria in either strategy and are indicated by a pink node with text indicating the ICD version(s) to which it is unique. Light Blue Nodes: ICD-9, Dark Blue Nodes: ICD-10, and Yellow Nodes: ICD-11

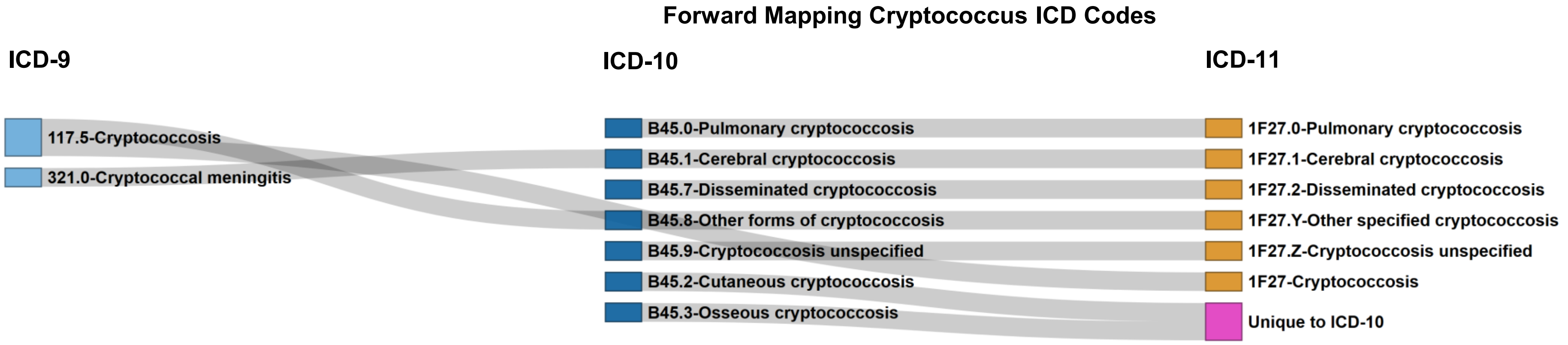

Supplemental Figure S9. Cryptococcus ICD Code Mapping: Best Match Forward Sankey Network
Nodes represent individual ICD codes across versions (ICD-9, -10, -11), connected by links indicating mapping pairs with the highest match score, with ties. Primary mapping utilized a stepwise strategy requiring a minimum cumulative match score of 45 out of 100 points. To capture complex relationships, a "skip-link" strategy was employed for pairs that either failed the initial threshold or exceed the stepwise score by ≥ 10 points. Codes with unlinked nodes ("Orphan" codes) represent version-specific entries that did not meet the minimum match criteria in either strategy and are indicated by a pink node with text indicating the ICD version(s) to which it is unique. Light Blue Nodes: ICD-9, Dark Blue Nodes: ICD-10, and Yellow Nodes: ICD-11

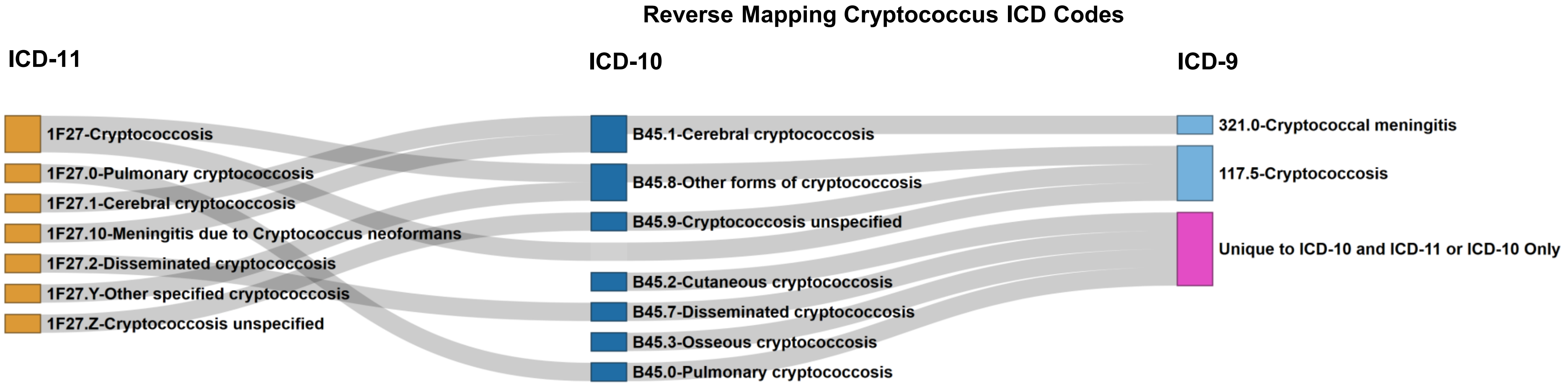

Supplemental Figure S10. Cryptococcus ICD Code Mapping: Best Match Reverse Sankey Network
Nodes represent individual ICD codes across versions (ICD-11, -10, -9), connected by links indicating mapping pairs with the highest match score, with ties. Primary mapping utilized a stepwise strategy requiring a minimum cumulative match score of 45 out of 100 points. To capture complex relationships, a "skip-link" strategy was employed for pairs that either failed the initial threshold or exceed the stepwise score by ≥ 10 points. Codes with unlinked nodes ("Orphan" codes) represent version-specific entries that did not meet the minimum match criteria in either strategy and are indicated by a pink node with text indicating the ICD version(s) to which it is unique. Light Blue Nodes: ICD-9, Dark Blue Nodes: ICD-10, and Yellow Nodes: ICD-11

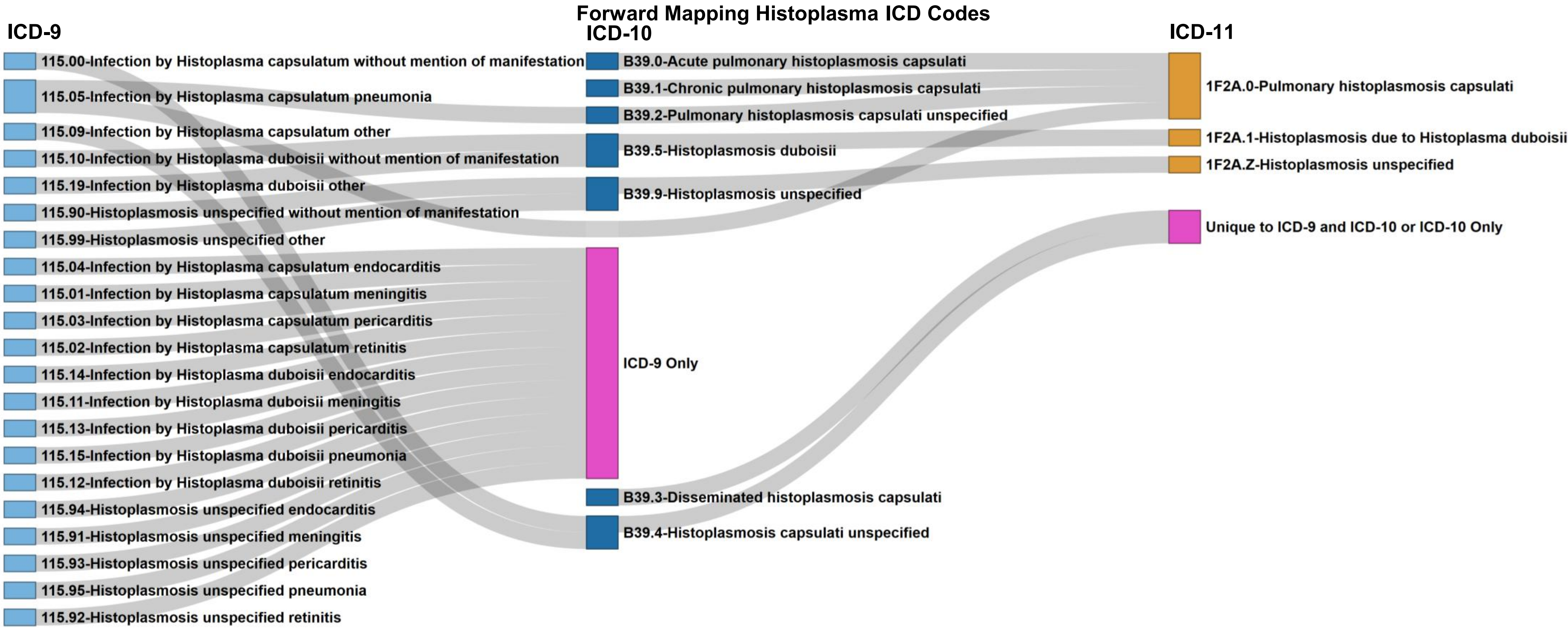

Supplemental Figure S11. Histoplasma ICD Code Mapping: Best Match Forward Sankey Network

Nodes represent individual ICD codes across versions (ICD-9, -10, -11), connected by links indicating mapping pairs with the highest match score, with ties. Primary mapping utilized a stepwise strategy requiring a minimum cumulative match score of 45 out of 100 points. To capture complex relationships, a "skip-link" strategy was employed for pairs that either failed the initial threshold or exceed the stepwise score by ≥ 10 points. Codes with unlinked nodes ("Orphan" codes) represent version-specific entries that did not meet the minimum match criteria in either strategy and are indicated by a pink node with text indicating the ICD version(s) to which it is unique. Light Blue Nodes: ICD-9, Dark Blue Nodes: ICD-10, and Yellow Nodes: ICD-11

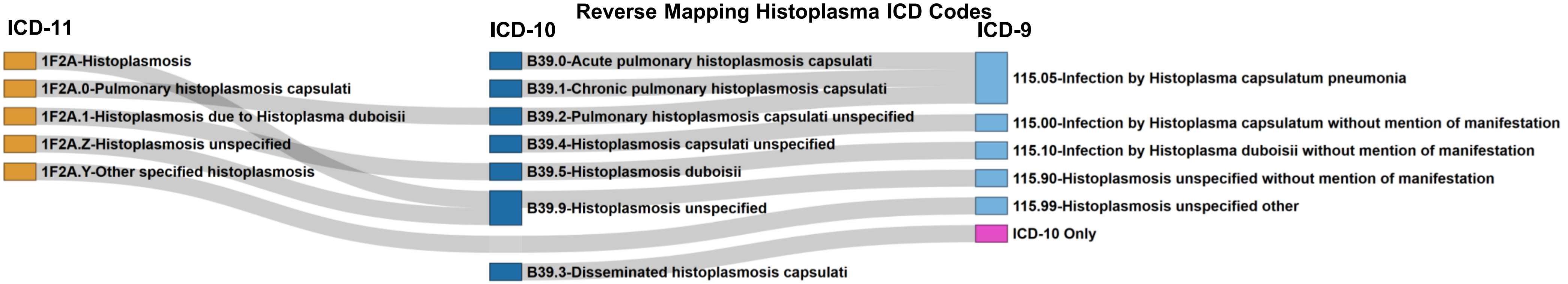

Supplemental Figure S12. Histoplasma ICD Code Mapping: Best Match Reverse Sankey Network

Nodes represent individual ICD codes across versions (ICD-11, -10, -9), connected by links indicating mapping pairs with the highest match score, with ties. Primary mapping utilized a stepwise strategy requiring a minimum cumulative match score of 45 out of 100 points. To capture complex relationships, a "skip-link" strategy was employed for pairs that either failed the initial threshold or exceed the stepwise score by ≥ 10 points. Codes with unlinked nodes ("Orphan" codes) represent version-specific entries that did not meet the minimum match criteria in either strategy and are indicated by a pink node with text indicating the ICD version(s) to which it is unique. Light Blue Nodes: ICD-9, Dark Blue Nodes: ICD-10, and Yellow Nodes: ICD-11

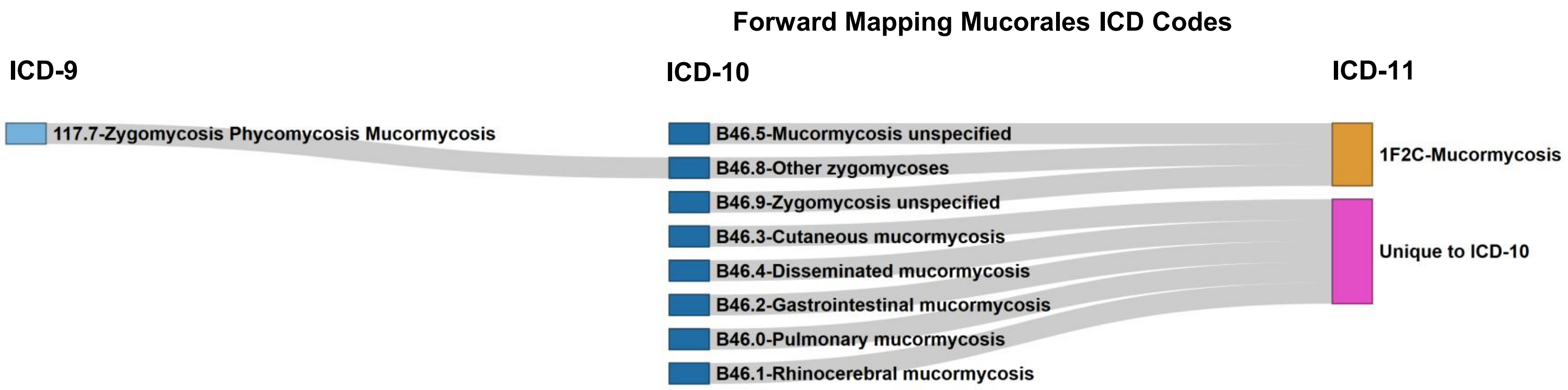

Supplemental Figure S13. Mucorales ICD Code Mapping: Best Match Forward Sankey Network
Nodes represent individual ICD codes across versions (ICD-9, -10, -11), connected by links indicating mapping pairs with the highest match score, with ties. Primary mapping utilized a stepwise strategy requiring a minimum cumulative match score of 45 out of 100 points. To capture complex relationships, a “skip-link” strategy was employed for pairs that either failed the initial threshold or exceed the stepwise score by ≥ 10 points. Codes with unlinked nodes (“Orphan” codes) represent version-specific entries that did not meet the minimum match criteria in either strategy and are indicated by a pink node with text indicating the ICD version(s) to which it is unique. Light Blue Nodes: ICD-9, Dark Blue Nodes: ICD-10, and Yellow Nodes: ICD-11

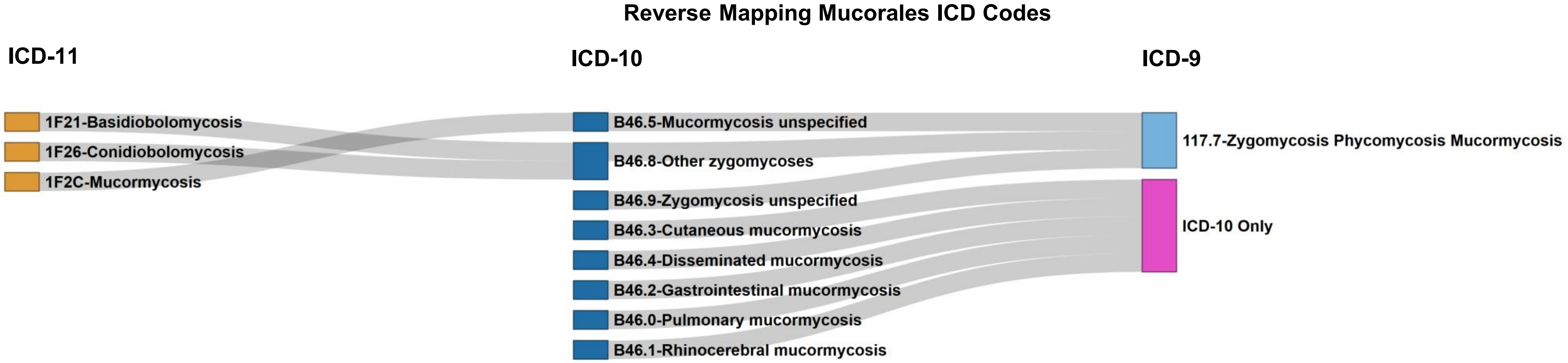

Supplemental Figure S14. Mucorales ICD Code Mapping: Best Match Reverse Sankey Network
Nodes represent individual ICD codes across versions (ICD-11, -10, -9), connected by links indicating mapping pairs with the highest match score, with ties. Primary mapping utilized a stepwise strategy requiring a minimum cumulative match score of 45 out of 100 points. To capture complex relationships, a “skip-link” strategy was employed for pairs that either failed the initial threshold or exceed the stepwise score by ≥ 10 points. Codes with unlinked nodes (“Orphan” codes) represent version-specific entries that did not meet the minimum match criteria in either strategy and are indicated by a pink node with text indicating the ICD version(s) to which it is unique. Light Blue Nodes: ICD-9, Dark Blue Nodes: ICD-10, and Yellow Nodes: ICD-11

Forward Mapping Paracoccidioides ICD Codes

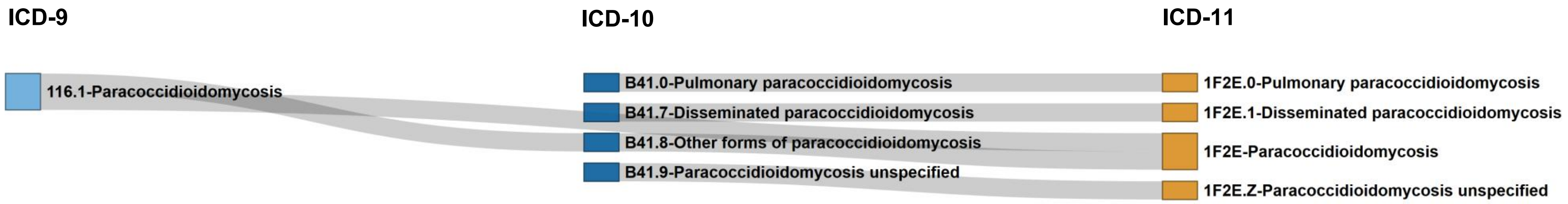

Supplemental Figure S15. Paracoccidioides ICD Code Mapping: Best Match Forward Sankey Network
Nodes represent individual ICD codes across versions (ICD-9, -10, -11), connected by links indicating mapping pairs with the highest match score, with ties. Primary mapping utilized a stepwise strategy requiring a minimum cumulative match score of 45 out of 100 points. To capture complex relationships, a "skip-link" strategy was employed for pairs that either failed the initial threshold or exceed the stepwise score by ≥ 10 points. Codes with unlinked nodes ("Orphan" codes) represent version-specific entries that did not meet the minimum match criteria in either strategy and are indicated by a pink node with text indicating the ICD version(s) to which it is unique. Light Blue Nodes: ICD-9, Dark Blue Nodes: ICD-10, and Yellow Nodes: ICD-11

Reverse Mapping Paracoccidioides ICD Codes

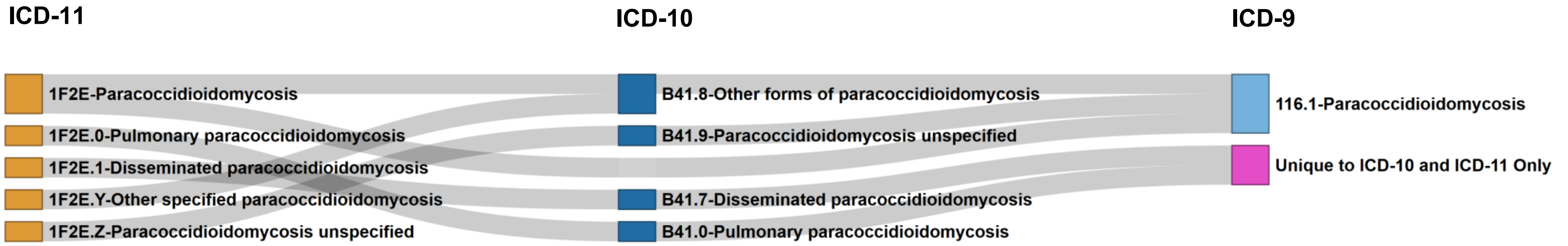

Supplemental Figure S16. Paracoccidioides ICD Code Mapping: Best Match Reverse Sankey Network
Nodes represent individual ICD codes across versions (ICD-11, -10, -9), connected by links indicating mapping pairs with the highest match score, with ties. Primary mapping utilized a stepwise strategy requiring a minimum cumulative match score of 45 out of 100 points. To capture complex relationships, a "skip-link" strategy was employed for pairs that either failed the initial threshold or exceed the stepwise score by ≥ 10 points. Codes with unlinked nodes ("Orphan" codes) represent version-specific entries that did not meet the minimum match criteria in either strategy and are indicated by a pink node with text indicating the ICD version(s) to which it is unique. Light Blue Nodes: ICD-9, Dark Blue Nodes: ICD-10, and Yellow Nodes: ICD-11

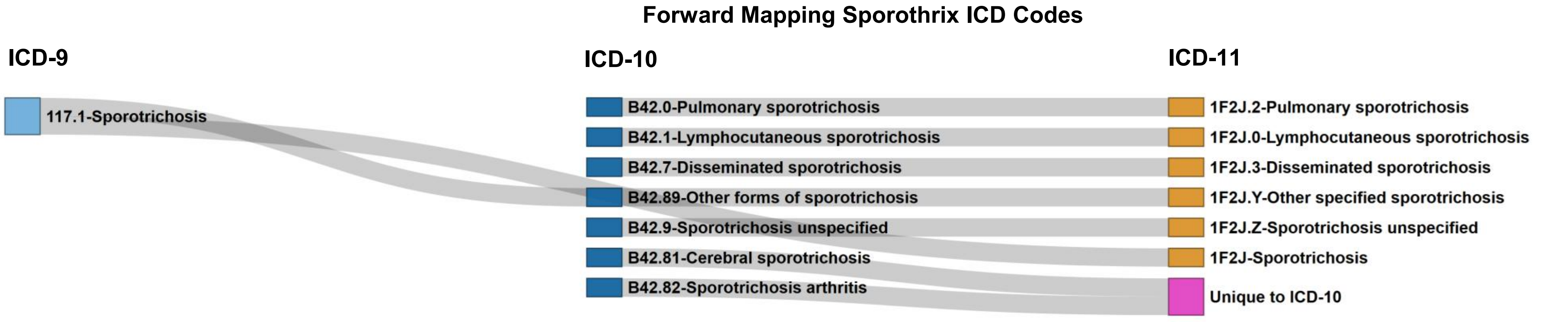

Supplemental Figure S17. Sporothrix ICD Code Mapping: Best Match Forward Sankey Network
Nodes represent individual ICD codes across versions (ICD-9, -10, -11), connected by links indicating mapping pairs with the highest match score, with ties. Primary mapping utilized a stepwise strategy requiring a minimum cumulative match score of 45 out of 100 points. To capture complex relationships, a “skip-link” strategy was employed for pairs that either failed the initial threshold or exceed the stepwise score by ≥ 10 points. Codes with unlinked nodes (“Orphan” codes) represent version-specific entries that did not meet the minimum match criteria in either strategy and are indicated by a pink node with text indicating the ICD version(s) to which it is unique. Light Blue Nodes: ICD-9, Dark Blue Nodes: ICD-10, and Yellow Nodes: ICD-11

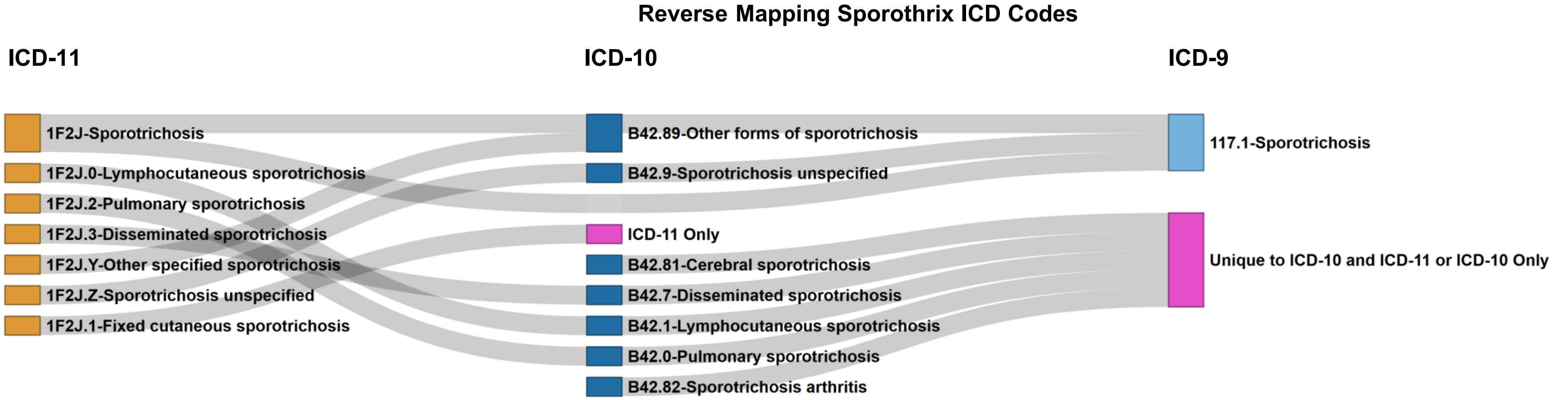

Supplemental Figure S18. Sporothrix ICD Code Mapping: Best Match Reverse Sankey Network
Nodes represent individual ICD codes across versions (ICD-11, -10, -9), connected by links indicating mapping pairs with the highest match score, with ties. Primary mapping utilized a stepwise strategy requiring a minimum cumulative match score of 45 out of 100 points. To capture complex relationships, a “skip-link” strategy was employed for pairs that either failed the initial threshold or exceed the stepwise score by ≥ 10 points. Codes with unlinked nodes (“Orphan” codes) represent version-specific entries that did not meet the minimum match criteria in either strategy and are indicated by a pink node with text indicating the ICD version(s) to which it is unique. Light Blue Nodes: ICD-9, Dark Blue Nodes: ICD-10, and Yellow Nodes: ICD-11

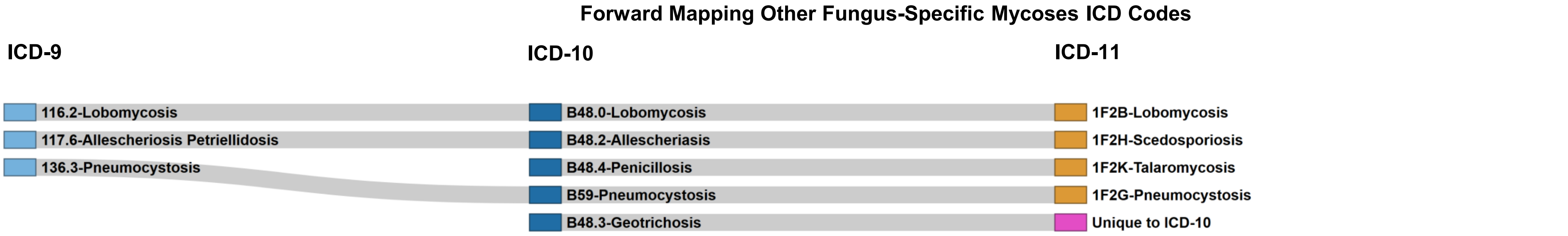

Supplemental Figure S19. Other Fungus-specific Mycoses ICD Code Mapping: Best Match Forward Sankey Network
Nodes represent individual ICD codes across versions (ICD-9, -10, -11), connected by links indicating mapping pairs with the highest match score, with ties. Primary mapping utilized a stepwise strategy requiring a minimum cumulative match score of 45 out of 100 points. To capture complex relationships, a “skip-link” strategy was employed for pairs that either failed the initial threshold or exceed the stepwise score by ≥ 10 points. Codes with unlinked nodes (“Orphan” codes) represent version-specific entries that did not meet the minimum match criteria in either strategy and are indicated by a pink node with text indicating the ICD version(s) to which it is unique. Light Blue Nodes: ICD-9, Dark Blue Nodes: ICD-10, and Yellow Nodes: ICD-11

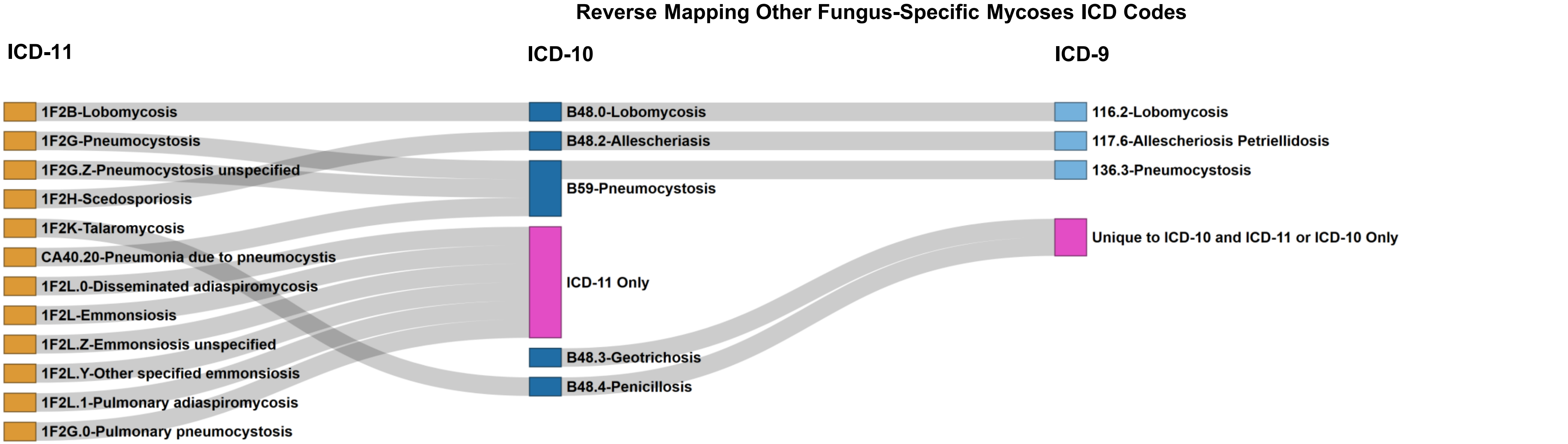

Supplemental Figure S20. Other Fungus-specific Mycoses ICD Code Mapping: Best Match Reverse Sankey Network
Nodes represent individual ICD codes across versions (ICD-11, -10, -9), connected by links indicating mapping pairs with the highest match score, with ties. Primary mapping utilized a stepwise strategy requiring a minimum cumulative match score of 45 out of 100 points. To capture complex relationships, a “skip-link” strategy was employed for pairs that either failed the initial threshold or exceed the stepwise score by ≥ 10 points. Codes with unlinked nodes (“Orphan” codes) represent version-specific entries that did not meet the minimum match criteria in either strategy and are indicated by a pink node with text indicating the ICD version(s) to which it is unique. Light Blue Nodes: ICD-9, Dark Blue Nodes: ICD-10, and Yellow Nodes: ICD-11

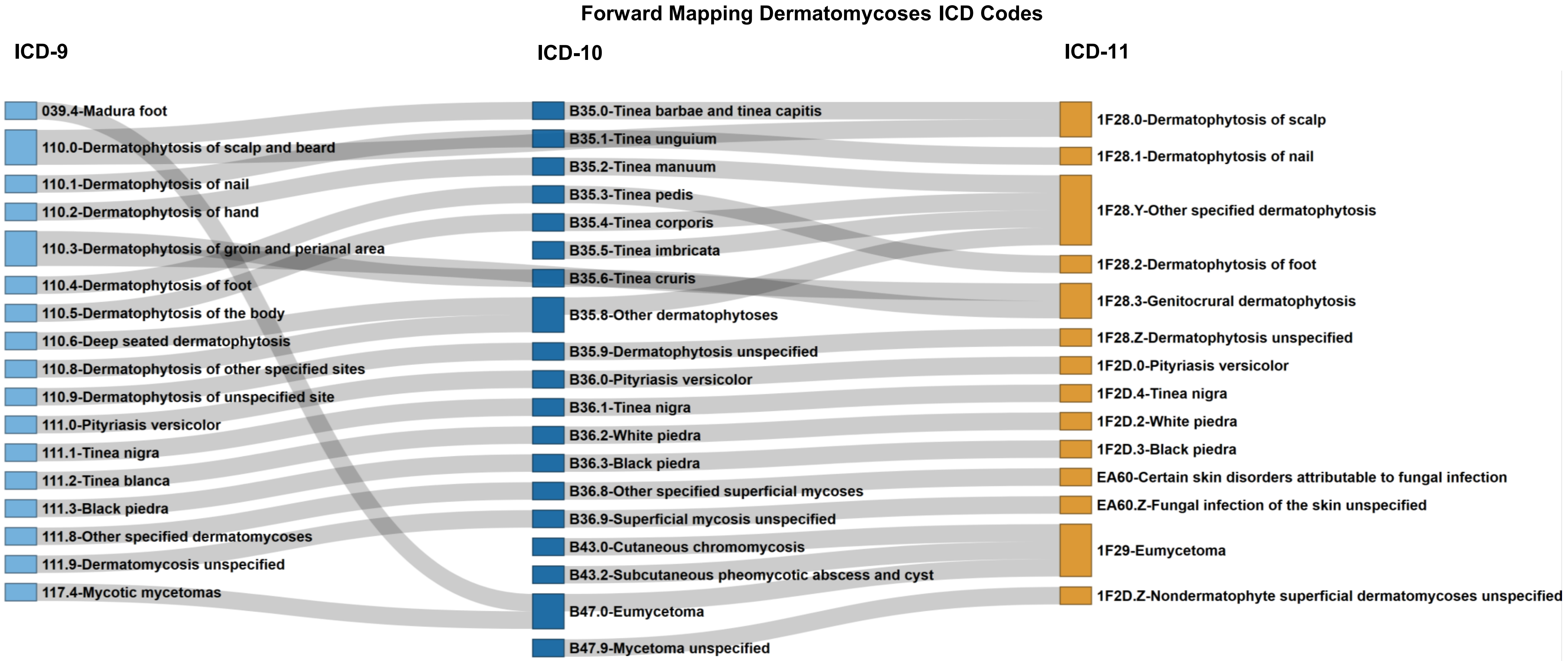

Supplemental Figure S21. Dermatomycoses ICD Code Mapping: Best Match Forward Sankey Network

Nodes represent individual ICD codes across versions (ICD-9, -10, -11), connected by links indicating mapping pairs with the highest match score, with ties. Primary mapping utilized a stepwise strategy requiring a minimum cumulative match score of 45 out of 100 points. To capture complex relationships, a “skip-link” strategy was employed for pairs that either failed the initial threshold or exceed the stepwise score by ≥ 10 points. Codes with unlinked nodes (“Orphan” codes) represent version-specific entries that did not meet the minimum match criteria in either strategy and are indicated by a pink node with text indicating the ICD version(s) to which it is unique. Light Blue Nodes: ICD-9, Dark Blue Nodes: ICD-10, and Yellow Nodes: ICD-11

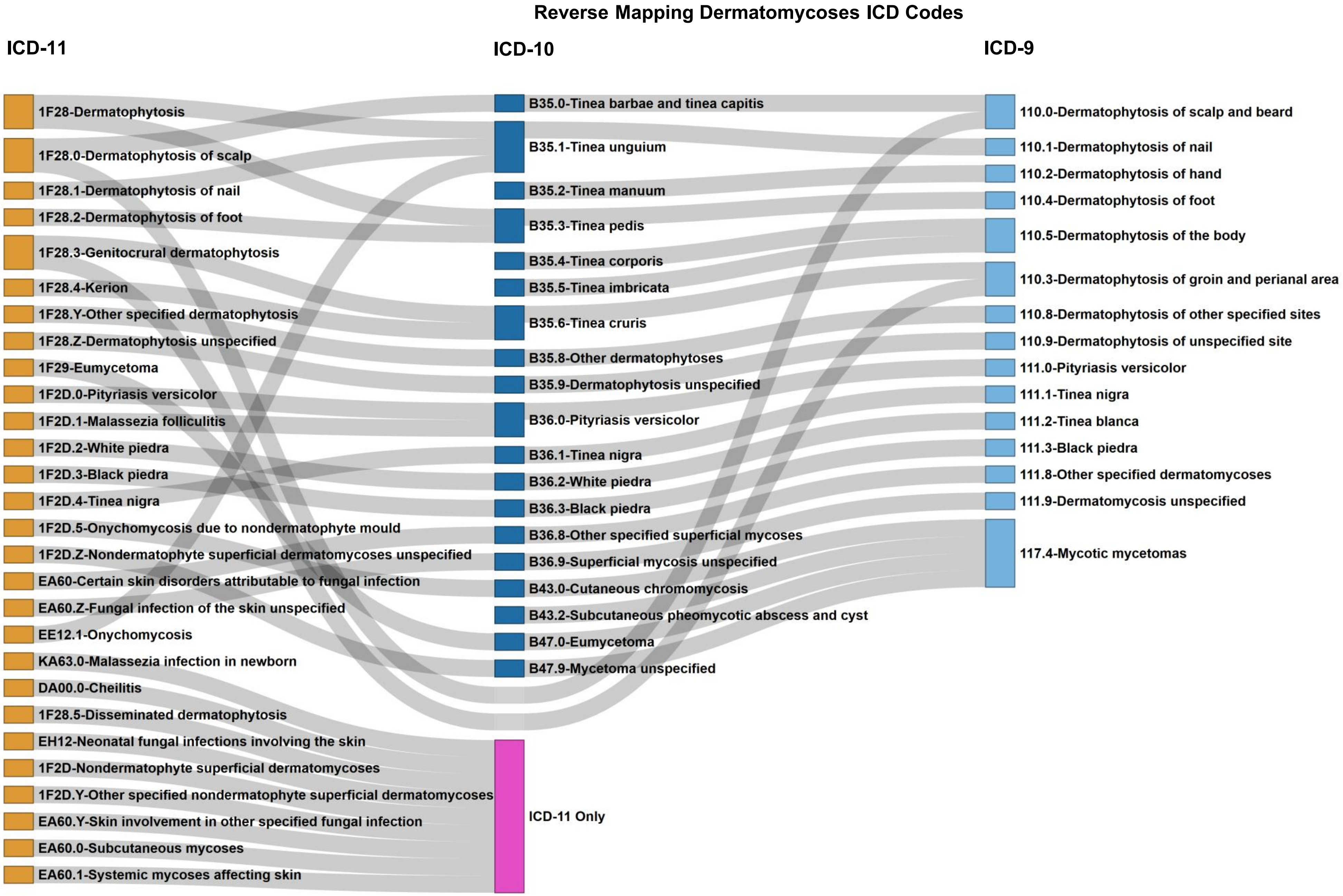

Supplemental Figure S22. Dermatomycoses ICD Code Mapping: Best Match Reverse Sankey Network
Nodes represent individual ICD codes across versions (ICD-11, -10, -9), connected by links indicating mapping pairs with the highest match score, with ties. Primary mapping utilized a stepwise strategy requiring a minimum cumulative match score of 45 out of 100 points. To capture complex relationships, a "skip-link" strategy was employed for pairs that either failed the initial threshold or exceed the stepwise score by ≥ 10 points. Codes with unlinked nodes ("Orphan" codes) represent version-specific entries that did not meet the minimum match criteria in either strategy and are indicated by a pink node with text indicating the ICD version(s) to which it is unique. Light Blue Nodes: ICD-9, Dark Blue Nodes: ICD-10, and Yellow Nodes: ICD-11

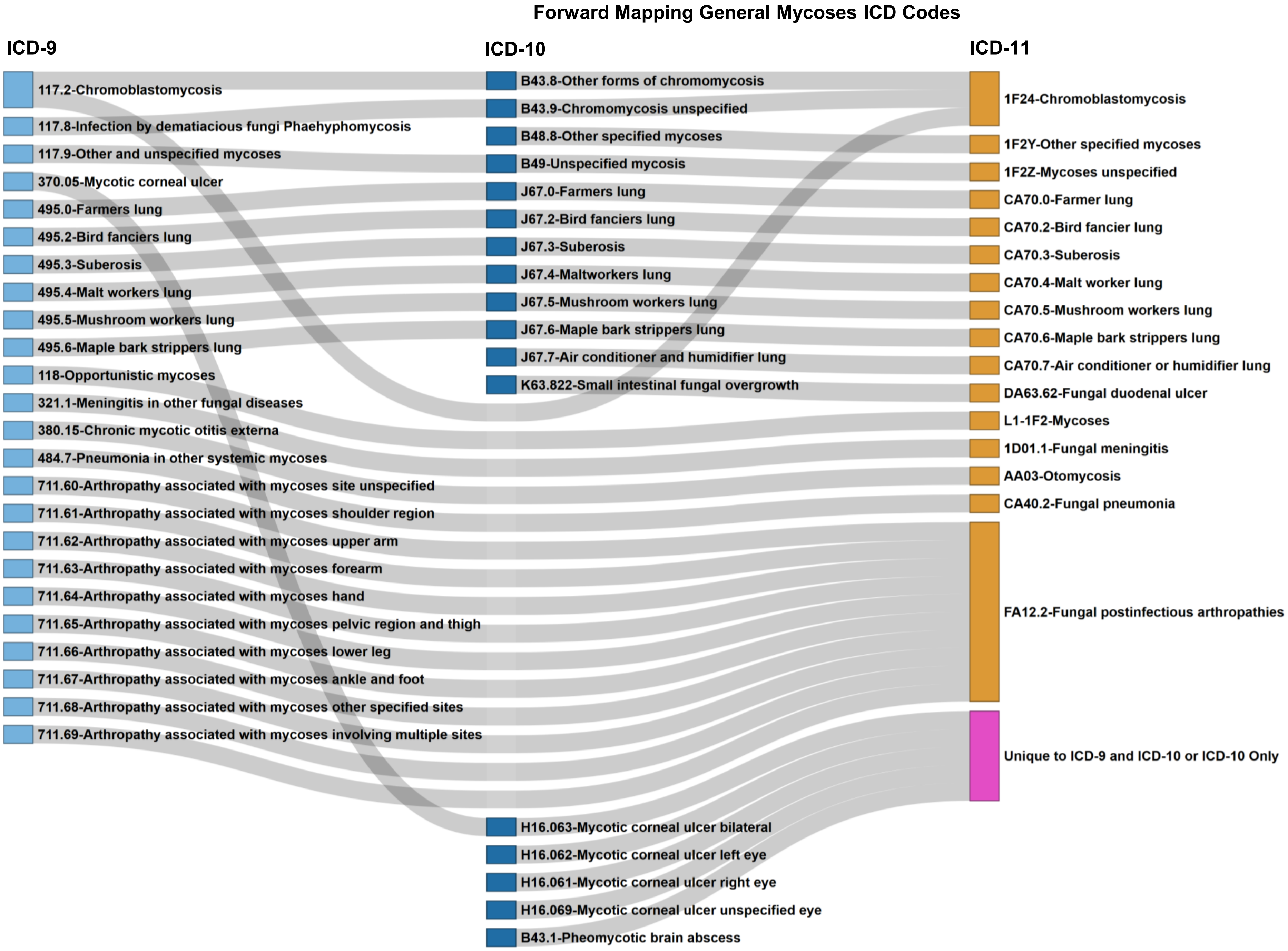

Supplemental Figure S23. General Mycoses ICD Code Mapping: Best Match Forward Sankey Network
Nodes represent individual ICD codes across versions (ICD-9, -10, -11), connected by links indicating mapping pairs with the highest match score, with ties. Primary mapping utilized a stepwise strategy requiring a minimum cumulative match score of 45 out of 100 points. To capture complex relationships, a "skip-link" strategy was employed for pairs that either failed the initial threshold or exceed the stepwise score by ≥ 10 points. Codes with unlinked nodes ("Orphan" codes) represent version-specific entries that did not meet the minimum match criteria in either strategy and are indicated by a pink node with text indicating the ICD version(s) to which it is unique. Light Blue Nodes: ICD-9, Dark Blue Nodes: ICD-10, and Yellow Nodes: ICD-11

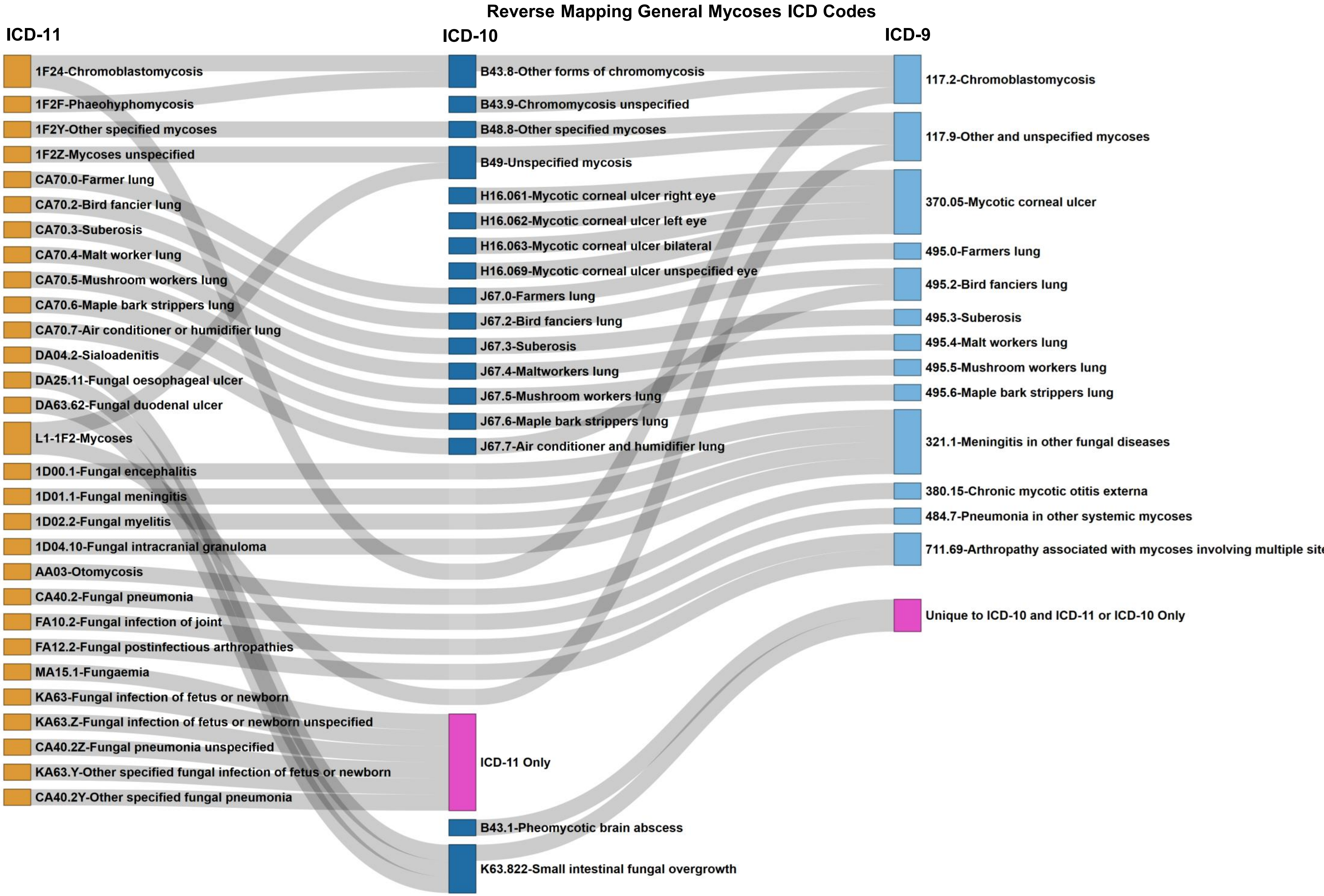

Supplemental Figure S24. General Mycoses ICD Code Mapping: Best Match Reverse Sankey Network
Nodes represent individual ICD codes across versions (ICD-11, -10, -9), connected by links indicating mapping pairs with the highest match score, with ties. Primary mapping utilized a stepwise strategy requiring a minimum cumulative match score of 45 out of 100 points. To capture complex relationships, a "skip-link" strategy was employed for pairs that either failed the initial threshold or exceed the stepwise score by ≥ 10 points. Codes with unlinked nodes ("Orphan" codes) represent version-specific entries that did not meet the minimum match criteria in either strategy and are indicated by a pink node with text indicating the ICD version(s) to which it is unique. Light Blue Nodes: ICD-9, Dark Blue Nodes: ICD-10, and Yellow Nodes: ICD-11
