# Supplementary material for: The Past and Future of Fungal Diagnoses in Health Records: A Comparative Analysis and Harmonization of Mycoses ICD Codes
Source: J Fungi (Basel). 2026 Oct 8;12(10):752. doi: 10.3390/jof12100752 (PMC13706049; doi:10.3390/jof12100752)
Supplement: Supplementary file 1 [file jof-12-00752-s001.zip › Supplemental_Methods_Final.pdf]

Systematic equivalence matching mycoses codes between ICD-9, ICD-10, and ICD-11: A resource for healthcare coding and EHR-based research

Marhiah C. Montoya[1], Brooke Esquivel[1], Mark A. Hoffman[2], Justin V. Remais[3], Theodore C. White[1]

[1] Division of Biological and Biomedical Systems; School of Science and Engineering; University of Missouri-Kansas City; Kansas City, MO 64110; USA
[2] Research Informatics; Children's Mercy Research Institute; Kansas City, MO 64108; USA
[3] Division of Environmental Health Sciences, University of California; Berkeley, CA 94720; USA

Corresponding authors:
Marhiah C. Montoya
Theodore C. White
University of Missouri – Kansas City
BSB198, 5007 Rockhill Rd.
Kansas City, MO. 64110
mmvmk@umkc.edu (M.C.M)
whitetc@umkc.edu (T.C.W)
816-235-2538

Supplemental Methods: Supplemental Methods of Analysis Pipeline

Data Sources and Software

Publicly available datasets containing the most recent iterations of each ICD version in the English language were obtained for this study through internet search. ICD-9 [16, 20] and ICD-10 [17, 21] code lists were accessed from the United States Centers for Medicare and Medicaid Services website (https://www.cms.gov/medicare/coordination-benefits-recovery/overview/icd-code-lists), as were the clinical modification version ICD-9-CM [18] (https://www.cms.gov/medicare/coding-billing/icd-10-codes/icd-9-cm-diagnosis-procedure-codes-abbreviated-and-full-code-titles) and ICD-10-CM [19] (https://www.cms.gov/medicare/coding-billing/icd-10-codes). ICD-11 code and description data were retrieved from the World Health Organization website (https://icd.who.int/browse/2025-01/mms/en) [3]. Data analysis and the creation of figures and tables were done using Microsoft Office Suite and R Studio. Original datasets, processed datasets, R code, and resulting figures and tables are accessible via GitHub repository link provided in the Supplementary Materials.

Identification of Fungal Disease Codes

ICD-9, ICD-10, and ICD-11 code datasets were systematically searched to identify diagnostic codes associated with fungal organisms or conditions with known fungal etiology. Codes were extracted and manually reviewed for study inclusion if they met one or more of the following criteria: 1) the code is located within the primary mycoses category; 2) the code description contained fungal-associated text including "fung-", "myco-", "mold", "mould", possessive suffixes (e.g., "-'s", "-s'"), or the first four letters of the genus name of any organism listed in the mycoses category; 3) the code described a condition known to have a fungal etiology, even if non-fungal causes were also possible. Clinical modification versions, ICD-9-CM and ICD-10-CM were also included in the analysis to identify fungal codes. Codes were excluded from further analysis if they met one or more of the following criteria: 1) the code was classified as a procedure code or extension code; 2) the code describes antifungal drug response or resistance, suspected exposure to a fungus, toxicity or poisoning due to mushroom ingestion, or an examination for a mycotic infection; and 3) the code describes a disease caused by a non-fungal organism

according to 2026 taxonomy. Included codes and descriptions were extracted from source files into a processed dataset and labeled according to ICD version of origin for downstream analysis.

Manual Systematic Description-Based Code Categorization for Match Score Calculation

Extracted codes were manually reviewed and classified as either taxon-specific mycoses or mycoses with unspecified or diverse fungal etiology. Taxon-specific ICD codes were classified into fungal groups based on the organism explicitly named in the code description, whereas codes with unspecified or diverse fungal etiologies were classified into fungal groups based on shared clinical themes. The number of codes within each fungal group was quantified and any taxon-specific fungal group containing five or more codes in at least one ICD version were classified as a main group, resulting in nine taxon-specific main groups. Taxon-specific fungal groups with fewer than five codes in any one ICD version were consolidated into a single main group, "Other Fungus-specific Mycoses", except for *Malassezia*-specific codes, which were consolidated within the "Dermatomycoses" main group alongside codes describing fungal skin conditions. Dermatomycoses consolidated codes from four different fungal groups including Malassezia-specific, dermatophyte, non-dermatophyte, and nonspecific dermatomycoses. Codes describing mycoses with unspecified or diverse fungal etiology were consolidated into one main group, "General Mycoses" containing nine fungal groups including five that were site-based (arthromycoses, neuromycoses, oculomycoses, otomycoses, pneumomycoses), two population-based categories (pediatric mycoses and occupation-related fungi), one fungal characteristic category (pigmented fungi), and a nonspecific fungal category. Both Dermatomycoses and General Mycoses fungal groups were classified as Mycoses with Diverse Fungal Etiology. After assignment to a specific fungal group and consolidation into one of twelve main groups, each code was further characterized by manual annotation of fungal order, genus, and species according to its current taxonomic classification or were assigned a value of "nonspecific" for codes with unspecified or diverse fungal etiology. Species-level information was only included if explicitly specified in code description text. In addition, three variables were created to capture clinical detail with respect to organ system affected, body site of disease manifestation, and if the condition was primarily internal, external, or nonspecific. Finally, a disease type variable was created to distinguish clinically meaningful subtypes within the same fungal group based on unique descriptive terms or themes. Following manual variable creation and code classification, the processed dataset was used for text mining-based description matching and match score calculation.

Description-based Text Mining and Match Score Calculation

ICD code descriptions were cleaned prior to text mining to reduce linguistic variability and naming inconsistencies. The cleaning process included standardizing terminology where required (e.g., "Maltworker's Lung" and "Malt Worker's Lung" were standardized to "Malt Worker's Lung"), stripping descriptions of punctuation, normalizing whitespace, converting text to lowercase, removing stop words, and alphabetizing text strings to minimize word order effects. Original description values were retained and cleaned text was stored as a new variable named final description. Unique comparison pairs were generated so that each ICD code description was compared to every other code description regardless of ICD version, producing 130,682 unique comparison pairs. Each comparison pair was evaluated across the manually curated taxonomic (fungal group, order, genus, species), clinical variables (affected organ system, body site, int-/externality, disease type), and final description. For each of these variables, exact matches were scored using a binary scheme (match = 1, mismatch = 0). To capture semantic similarity beyond exact text matching, fuzzy string similarity was calculated for the final description using the Jaccard distance method [22] with a q-gram of two which allowed for partial matches to be scored on a sliding scale ranging from 0 (no text overlap) to 1 (exact text string match). To facilitate cross-version mapping for fungal ICD codes based on these matching variables, we developed a weighted scoring

algorithm to quantify description-based similarity across ICD versions. The weighted scoring rubric was applied to the raw matches to reflect the relative importance of variables for clinically relevant cross-version mapping and had a maximum value of 100 points. Exact matches for the fungal group variable received the highest weight (25 points) to ensure organism-level matches were prioritized. Exact matches for fungal order, genus, and int-/externality did not have modified weights (1 point each), while exact species matches doubled in weight (2 points). Exact matches for affected organ system and disease type were weighed at 10 points each and body site had a weight of 15 points. Exact matches for final description were modified to a weight of 20 points. Finally, fuzzy description matches with a Jaccard similarity score ≥0.7 received an additional weight of 14 points plus the similarity score.

Bidirectional mapping of best matches and identification of version-specific ICD codes

To visualize cross-version ICD code matches, we developed a "Best Match" selection algorithm to identify the single strongest match, allowing for ties, and accounting for "orphan" codes that did not have a suitable match. To comprehensively assess code evolution and mycoses representation across ICD versions, the selection process was performed bidirectionally with forward going from ICD-9 to ICD-10 to ICD-11 and the reverse going from ICD-11 to ICD-10 to ICD-9. Bidirectional mapping was performed using three main steps: 1) taxonomic, ICD version, and match score filtering; 2) longitudinal skip-version mapping; and 3) orphan code identification.

The filtering step utilized a custom function to extract match pairs containing codes from different ICD versions within the same fungal group to prevent intra-version comparisons and taxonomically inappropriate matches. A minimum cumulative match score threshold of 45 points was applied to ensure sufficient match strength and pairs that failed to meet this threshold were excluded from standard stepwise mapping but later evaluated for potential alternative matches using a skip-link mapping strategy for comprehensive Sankey Network Diagram construction [23]. Within the filtered set of match pairs, best matches were selected based on the highest cumulative match score allowing for ties for accurate representation of one-to-many and many-to-one code matches across ICD versions. This standard stepwise strategy captured most cross-version code matches. To address match pairs that did not meet the 45-point threshold, we implemented a longitudinal skip-version (skip-link) mapping strategy allowing for match pairs directly from ICD-9 to ICD-11 in forward mapping or ICD-11 to ICD-9 in reverse mapping. Skip-link matches were also retained for pairs with match scores equal to or exceeding the stepwise match score by at least 10 points. This allowed individual codes to map directly to multiple versions when appropriate. Codes that did not meet the minimum cumulative match score threshold and were not included in either mapping strategy were classified as "orphan" codes, representing version-specific ICD codes. Following final review of all matches, Sankey network diagrams were generated to visualize cross-version relationships. Individual Sankey network diagrams for specific fungal groups are included in supplemental materials.

Data Availability Statement: All coding, data files, figures, and tables can be found at https://github.com/marhiahcmontoya/Fungal-ICD-Code-Mapping-Project

References

3. World Health Organization. ICD-11 - International Classification of Diseases 11th Revision - The global standard for diagnostic health information. Available online: https://icd.who.int/en/ (accessed on October 15, 2025).

16. United States Centers for Medicare and Medicaid Services. Valid ICD-9 List (XLSX). Available online: https://www.cms.gov/medicare/coordination-benefits-recovery/overview/icd-code-lists (accessed on October 15, 2025).

17. United States Centers for Medicare and Medicaid Services. Valid ICD-10 List (XLSX). Available online: https://www.cms.gov/medicare/coordination-benefits-recovery/overview/icd-code-lists (accessed on October 15, 2025).
18. United States Centers for Medicare and Medicaid Services. ICD-9-CM Diagnosis and Procedure Codes: Version 32 Full and Abbreviated Code Titles – Effective October 1, 2014 ZIP. Available online: https://www.cms.gov/medicare/coding-billing/icd-10-codes/icd-9-cm-diagnosis-procedure-codes-abbreviated-and-full-code-titles (accessed on October 15, 2025).
19. United States Centers for Medicare and Medicaid Services. 2026 ICD-10-CM & PCS Files April 1, 2026 Code Descriptions in Tabular Order (ZIP). Available online: https://www.cms.gov/medicare/coding-billing/icd-10-codes (accessed on October 15, 2025).
20. World Health Organization. International Classification of Diseases - Version 9 (ICD-9) Codes. Available online: https://www.cms.gov/medicare/coordination-benefits-recovery/overview/icd-code-lists (accessed on October 20, 2025).
21. World Health Organization. International Classification of Diseases - Version 10 (ICD-10) Codes. Available online: https://www.cms.gov/medicare/coordination-benefits-recovery/overview/icd-code-lists (accessed on October 20, 2025).
22. Levy, A.; Shalom, B.R.; Chalamish, M. A guide to similarity measures and their data science applications. *Journal of Big Data* **2025**, *12*, 188, doi:10.1186/s40537-025-01227-1.
23. Aviraj, K.S.; Mukhopadhyay, S.; Sharma, D.D.; Goyal, A.; Sagar, M.; Jose, J. Visualisation Approaches in Qualitative Metasynthesis for Health Research. *Cureus* **2026**, *18*, e101027, doi:10.7759/cureus.101027.
